# Influence of Hospital Outdoor Rest Space on the Eye Movement Measures and Self-Rating Restoration of Staff

**DOI:** 10.3389/fpubh.2022.855857

**Published:** 2022-03-16

**Authors:** Weiyi Cui, Zao Li, Xiaodong Xuan, Qingtao Li, Lei Shi, Xin Sun, Kai Zhu, Yi Shi

**Affiliations:** ^1^College of Architecture and Art, Hefei University of Technology, Hefei, China; ^2^Hefei Railway Engineering School, Hefei, China; ^3^Division of Life Sciences and Medicine, Department of Ophthalmology, The First Affiliated Hospital of University of USTC, University of Science and Technology of China, Hefei, China; ^4^Administration Department, The Second Affiliated Hospital of Bengbu Medical College, Bengbu, China; ^5^Neurosurgery Department, The Second Affiliated Hospital of Bengbu Medical College, Bengbu, China; ^6^Department of Emergency and Critical Care Medicine, The Third Affiliated Hospital of Anhui Medical University (The First People's Hospital of Hefei), Hefei, China

**Keywords:** hospital outdoor rest space, staff, eye movement measures, self-rating restoration, influence

## Abstract

**Objective:**

To investigate the effect of hospital outdoor rest space on the eye movement measures and self-rating restoration of staff.

**Background:**

Relieving the pressure of hospital staff through exposure to hospital outdoor rest space is essential, but there is a scarcity of research on the impact of hospital outdoor rest space on the eye movement measures and self-rating restoration of staff, especially for large Chinese hospitals.

**Methods:**

Cross-analysis was conducted based on the eye movement measures of 76 staff members obtained by eye movement tracking equipment in combination with the self-rating restoration scale and hospital outdoor rest space picture attributes (element proportion and position, brightness and saturation).

**Results:**

The differences in eye movement measures of different staff attributes (occupation, age, and gender) were identified, and the effects of hospital outdoor rest space picture attributes on the eye movement measures and self-rating restoration scale of staff were summarized. A number of proposals were also formulated: hospital outdoor rest space should be set up close to the working area of the group of medical staff; attention should be paid to the actual needs of senior staff members and the work pressure of junior nurses; the exposure to natural environment should be increased and the proportion of hard artificial elements should be reduced; the natural environment should be placed in the visual center; the saturation and brightness of hospital outdoor rest space should be increased; and staff members should have access to the sky environment in a variety of ways.

**Conclusion:**

The present study is an empirical study of evidence-based design on hospital outdoor rest space in China, and the results reveal the effects of hospital outdoor rest space on the eye movement measures and self-rating restoration of staff.

## Introduction

Stress is common in hospitals, especially for hospital staff who are experiencing a tense working environment (1). Notably, the role of environmental intervention in promoting medical quality has increased in prominence, and the focus of medical design has been expanded to care for the health and wellbeing of staff (2). Since exposure to the natural environment has a decompression effect on medical staff (3), the role of hospital outdoor rest space (HORS) is becoming more and more significant as the main carrier of the natural environment in the medical environment. Although hospital staff have minimal time to use HORS under heavy work pressure, even short-term contact with HORS during commuting could help staff obtain positive brain wave feedback and work pressure relief (4).

As revealed in previous studies, there are differences and relationships between the constituent elements of HORS on staffs' physiological brain wave feedback. Eye movement tracking technology could be used to objectively measure the gaze behavior of staffs and explain the characteristics of environmental recovery (5). Such technology is of considerable practical significance for exploring the eye movement measures (EMM) of staff in HORS, for improving the explanatory mechanism of HORS on staffs' restorative effects, and for guiding HORS design based on grasping the visual preferences of staff.

With the development of research in the field of restorative environment, two theories have gradually formed, stress reduction theory and attention restoration theory, and both have been adopted to make significant achievements in the research of the medical environment. Stress reduction theory holds that exposure to the natural environment could alleviate the physical, psychological and behavioral injuries caused by stressors (6), while attention restoration theory holds that individuals in a restorative environment could effectively restore their declining ability and attention (7). The development of theories related to the field of restorative environments has not only indicated the way for various types of research, but also laid the theoretical foundation for the development of restorative medical environment.

As well as providing a temporary perspective for staff (8), HORS could also improve the aesthetic and perceived value of hospital facilities (9). Notably, the motivation to create a restorative environment in HORS sometimes comes from staff, with the hope of alleviating the severe institutional environment and obtaining a quiet rest space (10). Thus, the support strategies involving mental health promotion activities in the medical environment should also consider the needs of staff (11).

At present, the focus of research on the restorative impact of HORS on staff has been on finding the purpose of HORS use for “fresh air” and the design preferences of nursing groups for HORS (12, 13). However, in such research, the main methods include questionnaires, interviews, and behavior observations (14, 15), and eye movement tracking technology is rarely used to explore the gaze behavior of staff in HORS at the level of physiological perception. Eye movement tracking technology has been used in numerous research fields, and is a suitable method for evaluating the visual perception of the built environment and could help people understand the complex characteristics of the restorative environment (5, 16).

Many achievements have been made in exploring the impact of the built environment on people's gaze behavior by using eye movement tracking technology. As an example, a flexible lawn combination has been found to improve the environmental visual attraction (17), the positive impact of landscape space elements on vision in urban leisure space (18), and the correlation between the proportion of different landscape space elements (17). In addition, the evaluation of green space attraction was found in combination with the questionnaire.

However, the use of eye movement tracking technology as a research method in restorative research is still relatively rare (5). Therefore, using eye movement tracking technology to investigate the effect of HORS on the EMM and self-rating restoration of hospital staff is not only an innovation in HORS recoverability research, but also an expansion of the research object. Through the collection of visual pictures and physiological data, and the use of analytical methods related to medical facilities, design characteristics could be further clarified (19).

Additionally, there is a lack of focus on Third-Level Tier 1 General Hospitals (T1GH), the highest-level hospitals in China, in terms of case selection. Different from the Western medical system, residents often directly choose T1GH with advantageous medical resources after illness due to the imperfect hierarchical diagnosis and treatment system, and the number of outpatient and emergency visits of millions of people per year has become the norm. Such high-intensity demand for medical treatment has also led to excessive tension among hospital staff (16), especially since there is minimal opportunity for recovery in the workplace, which would exacerbate the mental pressure of staff (20).

Therefore, the aims of the present study were to investigate the effects of HORS on the EMM and self-rating restoration of staff in large hospitals in China, to analyze the differences and commonalities among staff, combined with self-rating restoration scale (SRRS), and finally to propose optimization suggestions for HORS from the perspective of promoting the recoverability of staff.

## Study Aim and Hypothesis

To achieve the aforementioned aims, three specific hypotheses were proposed based on the attention restoration theory that observation of the outdoor environment has a restorative effect on energy-intensive activities such as work (21), and the stress reduction theory that exposure to the external environment could alleviate physical, psychological, and behavioral damage caused by stressors (6).

1. There are differences in EMM [time to first fixation (TFF), total fixation time (TFD), average pupil size (APS)] of staff with different attributes when watching HORS pictures;
2. The attributes of HORS pictures (proportion and position of elements, saturation and brightness of pictures) are related to the EMM of staff;
3. The attributes of HORS pictures (proportion and position of elements, saturation and brightness of pictures) are related to the scores of SRRS.

## Methods

### Sample Pictures Selection

Four T1GHs were selected for the study, which had all been newly built-in and were all located in Hefei, the capital city of Anhui province. Through field visits, the investigators identified 20 HORSs that were more typical of the four T1GH hospitals, and all sample pictures were taken between 12:00 and 14:00 on August 2 and 3, 2021 [Cloudy, 33°C (91.4°F)−25°C (77°F), northeast wind level 1–2], with a view height of 1.6 m (5.25 ft) and 120° view angle (Table 1) (22).

**Table 1:** 20 Sample pictures used in this research.

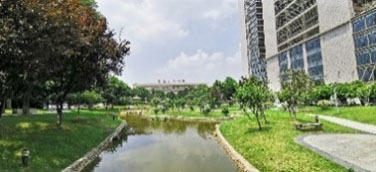	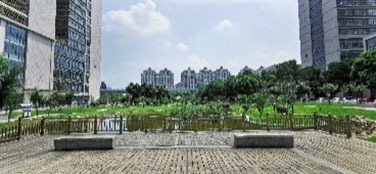	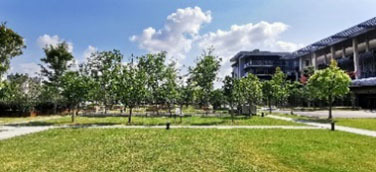	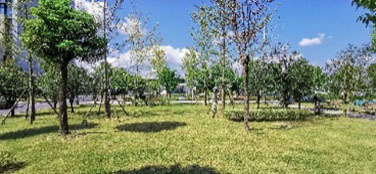
1	2	3	4
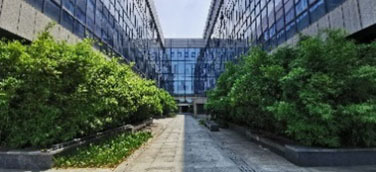	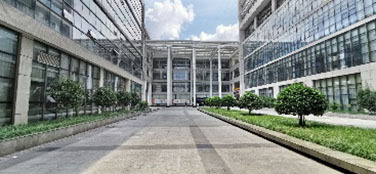	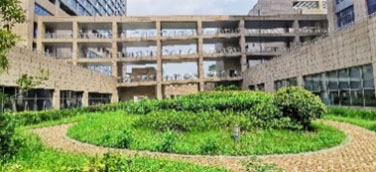	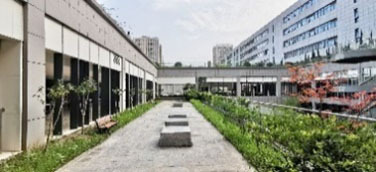
5	6	7	8
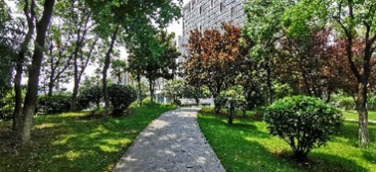	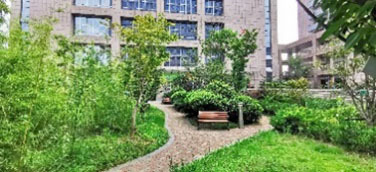	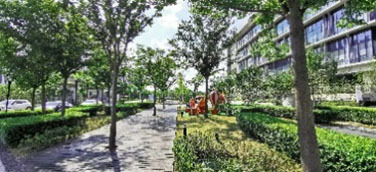	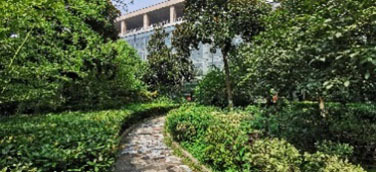
9	10	11	12
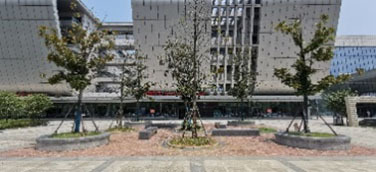	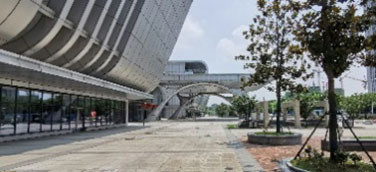	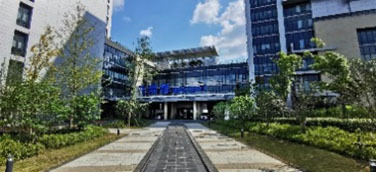	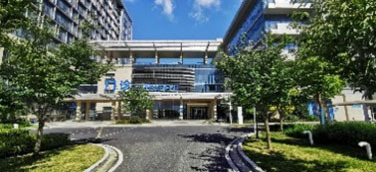
13	14	15	16
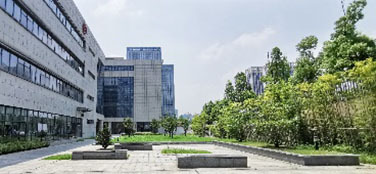	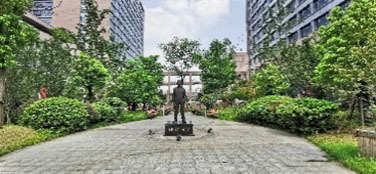	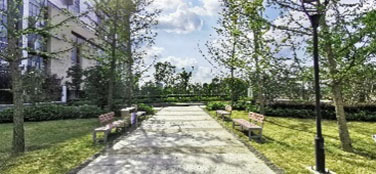	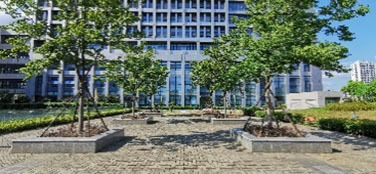
17	18	19	20

### Participants

To avoid possible data distortion due to the inherent views of the staff in the four T1GHs, Hospital BY was selected for the experiment, which is also a T1GH, and the approval of the hospital ethics committee was obtained. Before the experiment, all staff members were fully informed of the purpose and process of the experiment, and oral consent from all staff members was obtained.

The formal experiment was conducted between August 9 to 15, 2021, and a total of 90 staff members (30 each of doctors, nurses, and administrators) were recruited. After excluding 14 groups, of which the sampling rate was <80% or were interrupted by an emergency recall to participate in medical rescue, 76 valid data groups were finally obtained (Table 2).

**Table 2:** Attributes and proportion of staffs.

				**Unit: Number of people**					
**Gender**			**Age**			**Occupation**			**Average age**	**Male**	**Female**
Male	41	53.9%	Under 20	4	5.26%	Doctor	24	31.6%	35.83	22 (91.67%)	2 (8.33%)
Female	35	46.1%	21–30	31	40.79%	Nurse	27	35.5%	27.22	2 (7.41%)	25 (92.59%)
			31–40	28	36.84%	Administrator	25	32.9%	36.72	17 (68.00%)	8 (32.00%)
			41–50	6	7.89%						
			51–60	7	9.21%						

By comparing the gender and age of the three occupational groups, the average age of the administrator group (36.72) was found to be the largest, followed by the doctor (35.83) and nurse (27.22) groups. For the gender distribution characteristics, males accounted for the largest proportion in the doctor group (22/24, 91.67%), followed by the administrator (17/25, 68%) and nurse groups [2/27 (7.41%)] (Table 2).

### Study Design and Procedures

**Figure 1:**
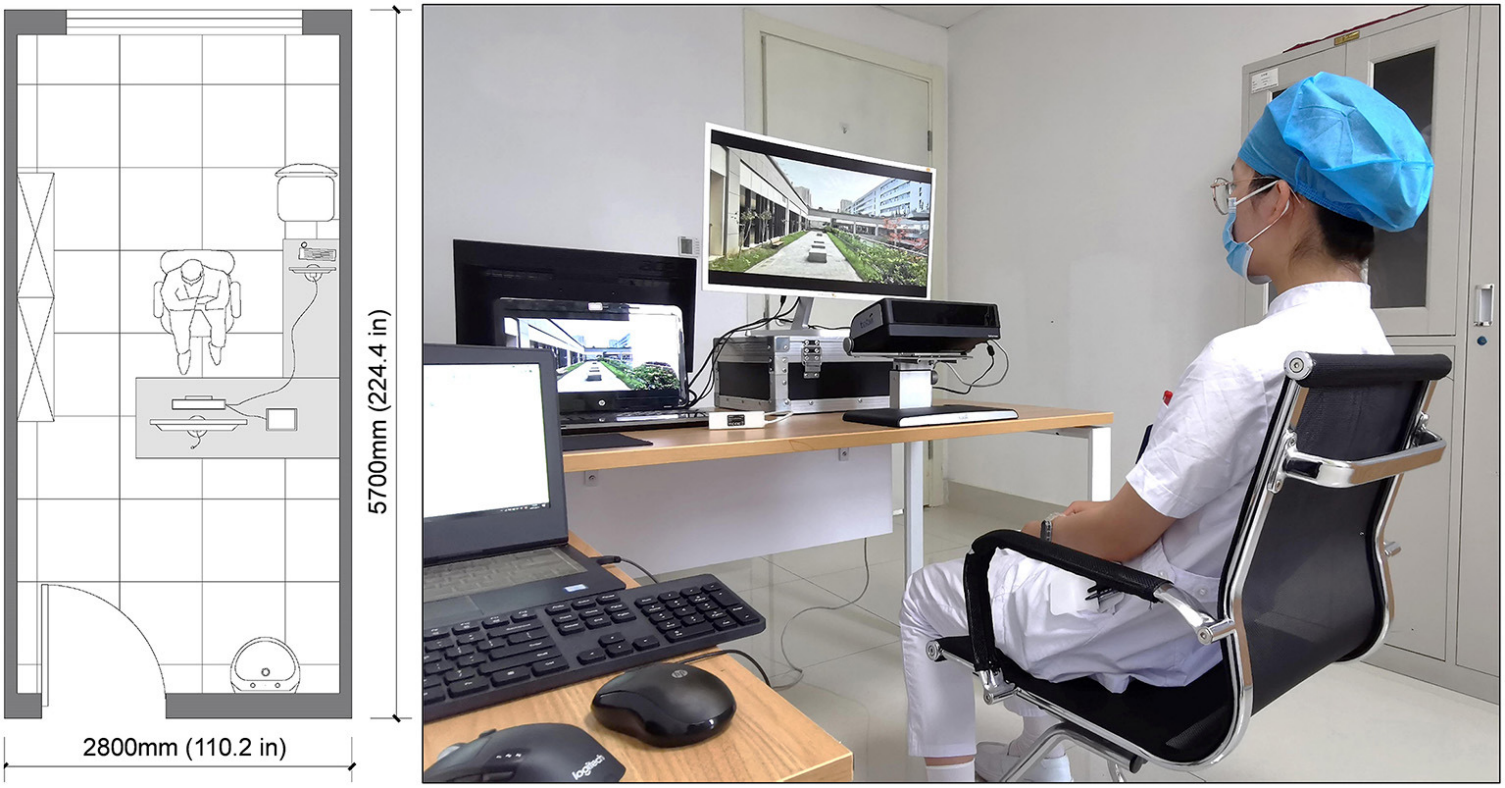
The arrangement of experimental site.

The experimental procedure was optimized based on relevant research experience in combination with the characteristics of the present research (23). The experiment was conducted by displaying 20 alternating sample pictures on a 27-inch display screen for 10 s each. After the experiment was completed, staff members were invited to continue answering the SRRS after a short break until the entire process was completed. The experimental site was arranged in a quiet office close to the staff workplace (Figure 1).

To ensure the reliability and effectiveness of the formal experiment, the researcher conducted pre-experiments on August 6, 2021, involving two hospital executives, one ophthalmologist, one neurosurgeon and two administrators to optimize the experimental process.

### Eye-Tracking Technology

In the present study, the Tobii T120 eye tracker (sampling rate 120 hz) was used to record EMM, and the area of interest (AOI) was divided and analyzed by Tobii Studio Software. In AOI analysis, 20 sample pictures were demarcated according to two categories and their contained space elements (sky, landscape, facade, and hard paving) to form AOI groups for the calculation of the EMM of four space elements (Table 3, Supplementary Material).

**Table 3:** AOIs, geometrical center points and fixation diagram of sample picture.

AOIs of sample picture	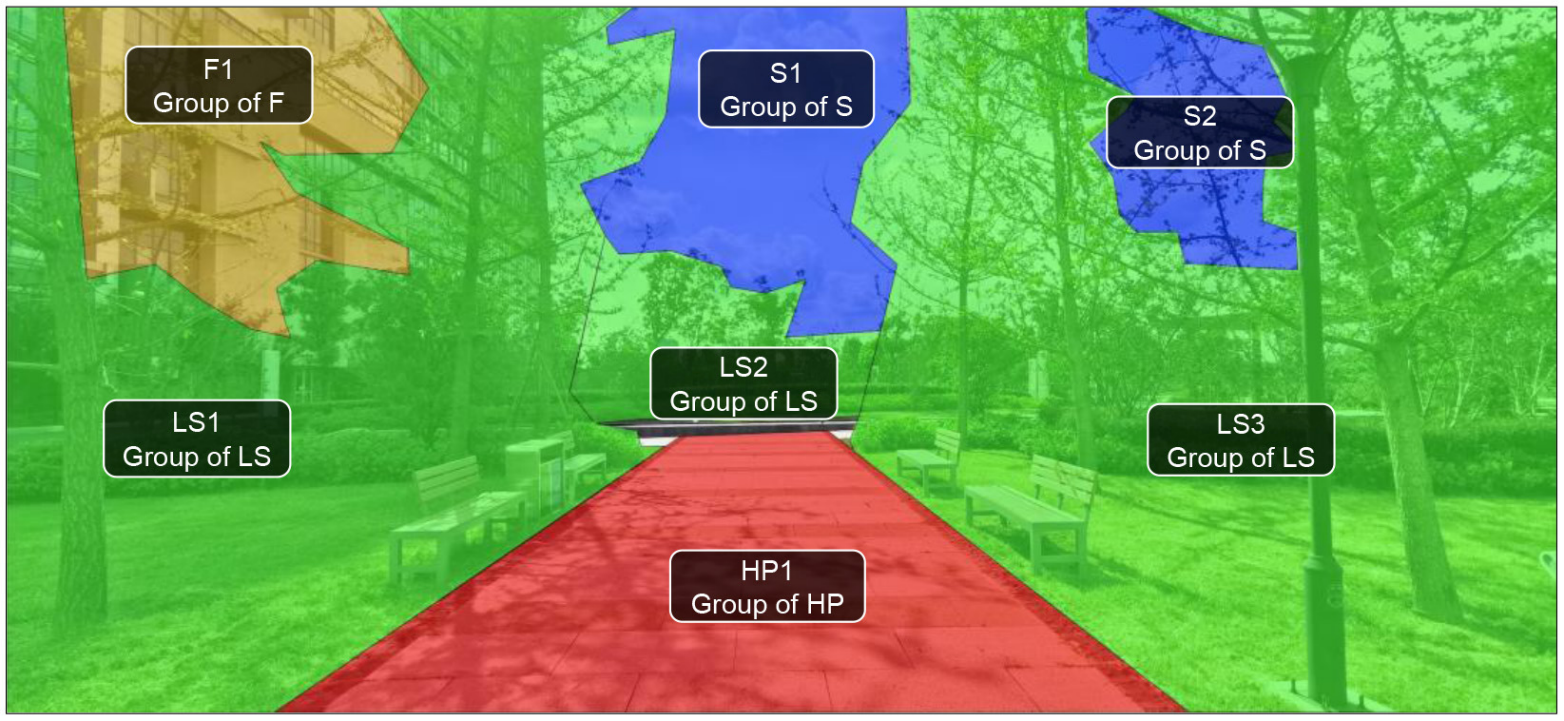
Geometrical center point of each AOI in sample picture	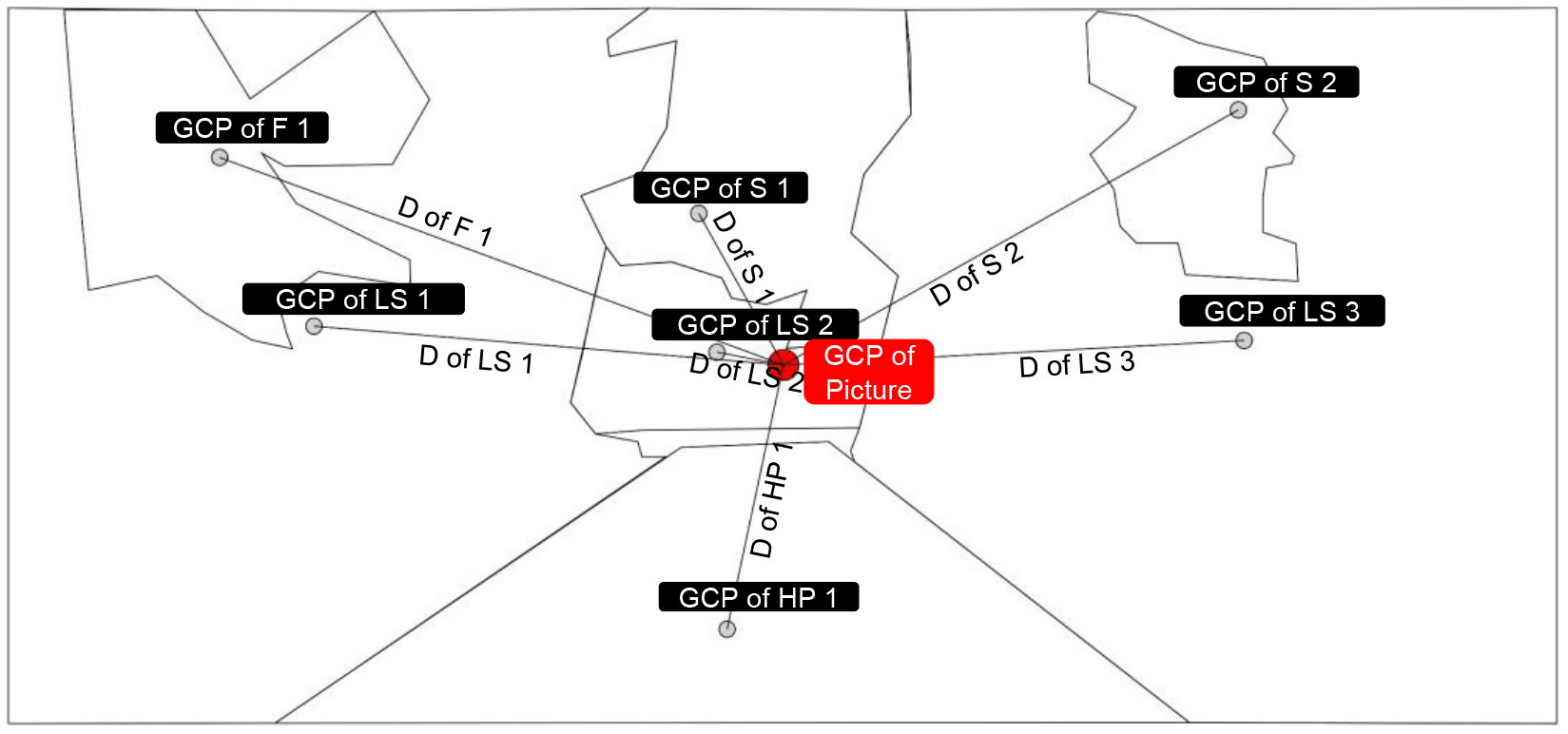
Fixation diagram of sample picture	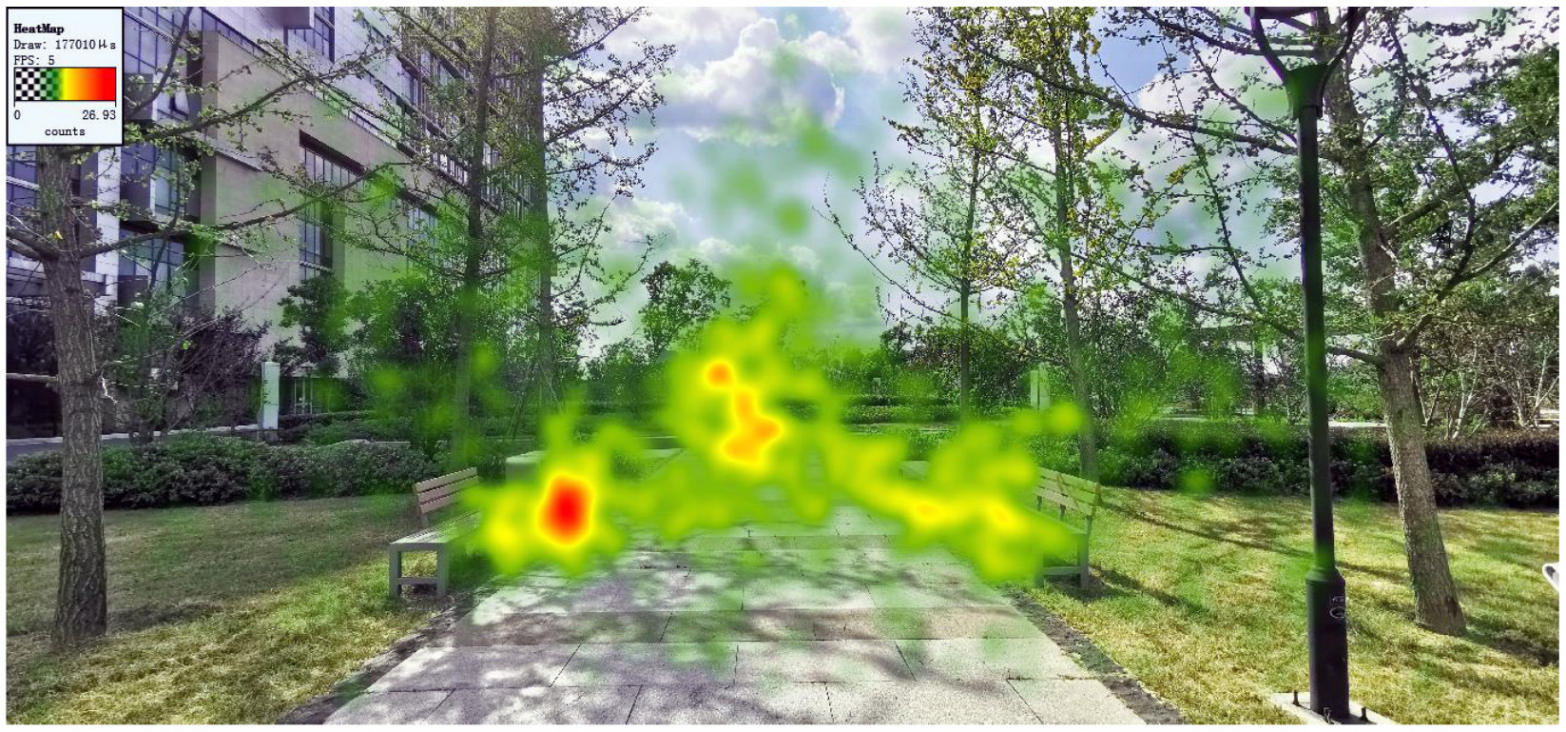

*LS, Landscape Space Element; S, Sky Element; F, Facade Element; HP, Hard Paving Element; GCP, Geometrical Center point; D, Distance*.

Based on defining AOI, three eye movement measures (EMM) commonly used in eye movement tracking technology were selected to explore the visual preferences of staffs: (1) TFF, which indicates the priority of the staffs' fixation on the elements in the picture (24, 25), with a shorter fixation indicating easier recognition (26); (2) TFD, which indicates the sum of the staffs' fixation duration of a certain element (24), with a longer duration indicating greater interest and participation (27, 28); and (3) APS, which could be used as a summative index of brain activity (29), and is related to the cognitive burden of staffs, with a greater cognitive burden indicating greater mental load (30).

### Properties of Sample Picture

To grasp the correlation between the EMM of staff and the proportion of space elements, firstly, four AOI groups (that is the four space elements: facade, sky, hard paving, and landscape) were outlined in each picture through drawing software (Photoshop CS6). The proportion of the number of pixels in each AOI groups to the total pixels of the picture was then calculated, as well as the saturation and brightness of the picture.

Secondly, each region of AOIs was described in the software (AutoCAD 2016) to identify the geometrical center point and the distance to the center point of the picture (CPP). Finally, the average distance between the geometrical center point (GCP) of each AOIs contained in an element to the CPP was used to represent the position of the element in the picture (Table 3).

### Self-Rating Restoration Scale

Since many of the existing restorative scales are based on attention restoration theory, with minimal consideration for stress reduction theory, and have the characteristics of obscure wording and long questionnaires, hospital staff who are not familiar with the relevant theories may become confused. Therefore, an improved SRRS was selected as the research tool. SRRS is a concise, clear, and easy to measure questionnaire composed of four dimensions of emotional, behavioral, cognitive, and physiological, and eight sub-items (31).

At present, SRRS has been applied in the Chinese context and has achieved high reliability and validity. Moreover, because the EMM investigated in the present study belongs to physiological evaluation, the physiological dimension and two sub-items were excluded in the used questionnaire to save the response time of the staff. The three dimensions of emotional, behavioral, and cognitive, and a total of six sub-items were retained.

## Results

A series of analysis through one-way ANOVA, independent sample *t*-test, Spearman's rank correlation and partial least squares regression was conducted by SPSS software (version 24.0, IBM Corporation, New York, USA), so as to explore the differences of EMM among staff with different attributes, and their relationship with SRRS and HORS picture attributes (element proportion and position, brightness and saturation).

### Correlation Between Staff Attributes and Eye Movement Measures

#### Differences of Eye Movement Measures Among Different Occupations

Through one-way ANOVA, significant differences were found among different occupations in the TFD of staff on the landscape space element (*p* = 0.022) and APS (*p* = 0.0001) (Table 4).

**Table 4:** One-way ANOVA of EMM between three occupations.

**Occupation**	**TFD of L (M ±SD)**	**APS (M ±SD)**
Doctor (*n* = 24)	75.44 ± 16.56	3.62 ± 0.69
Nurse (*n* = 27)	77.04 ± 15.51	3.90 ± 0.75
Administrator (*n* = 25)	63.64 ± 22.51	3.08 ± 0.47
Box plot	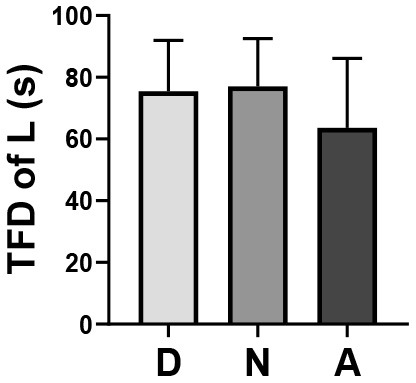	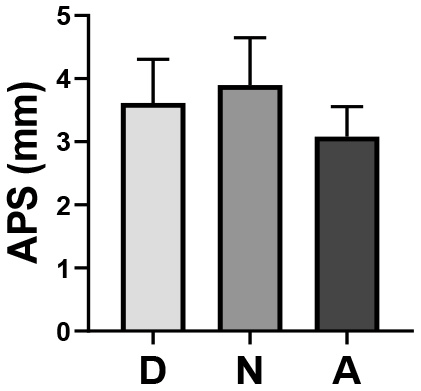
F	4.009	10.442
p	0.022^*^	0.0001^**^

* *p < 0.05*,

** *p < 0.01; TFD of LS, Total Fixation Duration of Landscape Space Element; APS, Average Pupil Size; M, Mean; SD, Standard Deviation*.

On the premise that the data met the variance homogeneity test, through Bonferroni correction for multiple comparisons, the difference of the TFD of staff on the landscape space element mainly existed between the nurse group and administrator group (*p* = 0.032), that is, the TFD of the nurse group on the landscape space element was higher than that of the administrator group. The difference in APS could be mainly attributed to the small size of the administrator group, that is, the APS of the administrator group was smaller than that of the doctor group (*p* = 0.016) and nurse group (*p* = 0.000) (Table 5).

**Table 5:** The Bonferroni correction for multiple comparisons for the difference of EMM between occupation.

**Dependent variable**				**Mean difference (I-J)**	**Std. error**	**Sig**.
Bonferroni	TFD of landscape space element	N	D	1.59958	5.16220	1.000
			A	13.39800^*^	5.10724	0.032
	Average pupil size	D	N	−0.28190	0.18250	0.380
			A	0.53385^*^	0.18590	0.016
		N	D	0.28190	0.18250	0.380
			A	0.81575^*^	0.18055	0.000

* *p < 0.05*,

*D, Doctor; N, Nurse; A, Administrator; TFD, Total Fixation Duration*.

#### Differences of Eye Movement Measures Between Gender

At the same time, through one-way ANOVA, a significant difference in APS was found between different genders (*p* = 0.020), that is, the APS of males was smaller than that of females (Table 6).

**Table 6:** One-way ANOVA of of average pupil size between gender.

**Gender**	**APS (M ±SD)**
Male (*n* = 41)	3.36 ± 0.73
Female (*n* = 35)	3.75 ± 0.68
Box plot	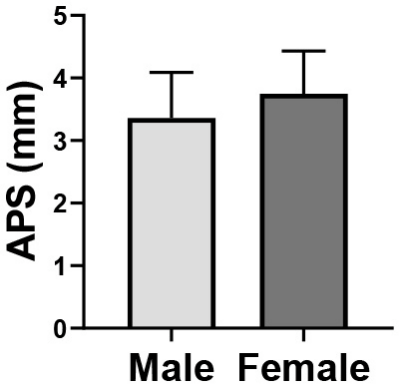
F	5.680
p	0.020^*^

* *p < 0.05*,

*M, Mean; SD, Standard Deviation*.

#### Correlations Between Staff Age and Eye Movement Measures

At the age level, through Spearman's rank correlation, negative correlations were found between staff age and APS (r = −0.504^**^, *p* = 0.000), and the TFD of staff on the landscape space element (r =-0.304^*^, *p* = 0.008). Meanwhile, positive correlations were found between staff age and the TFF of staff on the facade element (r = 0.242^*^, *p* = 0.035) and hard paving element (r = 0. 313^**^, *p* = 0.006) (Table 7).

**Table 7:** Correlations coefficient between age and EMM.

			**Average pupil size**	**TFD of landscape space element**	**TFF of facade element**	**TFF of hard paving element**
Spearman's rho	Age	Correlation coefficient	−0.504^**^	−0.304^**^	0.242^*^	0.313^**^
		Sig. (2-tailed)	0.000	0.008	0.035	0.006

* *Correlation is significant at the 0.05 level (2-tailed)*.

** *Correlation is significant at the 0.01 level (2-tailed)*.

*TFF, Time to First Fixation; TFD, Total Fixation Duration*.

### Correlation Analysis Between Space Attributes and Eye Movement Measures

#### Correlation Analysis Between Elements Proportion and Eye Movement Measures

In the present study, PLS, which is suitable for small sample sizes and may have collinearity, was selected as the analytical method. Based on the PLS regression analysis of the proportion of four space elements and three types of EMM of staff, the variable importance in projection >0.8 was selected (32), and the statistical regression model with the dependent variable was established.

Firstly, the APS of staff would be negatively affected by the proportion of sky element and positively affected by the proportion of hard paving element. Secondly, the TFD of staff on most constituent elements in HORS pictures would be positively affected by the proportion of itself and negatively affected by the proportion of elements belonging to different categories.

At the same time, the TFD of staff to the sky element was found to be negatively affected by the proportion of landscape space element and the proportion of facade element. Finally, the TTF of staff to each constituent element would be negatively affected by the proportion of natural environment and positively affected by the proportion of hard artificial elements (Table 8).

**Table 8:** Partial least squares regression between EMM and the proportion of space elements.

**Name of elements**		**P of LS**	**P of S**	**P of F**	**P of HP**
APS	VIP	0.069	**1.663**	0.651	**0.898**
	SRC	−0.013	−0.322	0.126	0.174
	APS = −0.322 × P of S+0.174 × P of HP				
TFD of HP	VIP	0.748	0.713	0.579	**1.612**
	SRC	−0.188	−0.179	0.145	0.405
	TFD of HP =0.405 × P of HP
TFD of F	VIP	**1.112**	0.599	**1.241**	**0.930**
	SRC	−0.278	−0.150	0.310	0.232
	TFD of F = −0.278 × P of LS+0.310 × P ofF+0.232 × P of HP				
TFD of L	VIP	**0.938**	0.767	**0.996**	**1.241**
	SRC	0.180	0.147	−0.191	−0.239
	TFD of L = 0.180 × P of LS−0.191 × P of F−0.239 × P of HP				
TFD of S	VIP	**1.373**	0.431	**1.386**	0.078
	SRC	−0.240	0.075	0.242	−0.014
	TFD of S = −0.240 × P of LS+0.242 × P of F				
TFF of HP	VIP	**1.263**	0.172	**1.158**	1.017
	SRC	−0.197	−0.027	0.181	0.159
	TFF of HP = −0.197 × P of LS+0.181 × P of F+0.159 × P of HP				
TFF of F	VIP	**0.993**	0.783	**1.161**	**1.026**
	SRC	−0.163	−0.129	0.191	0.168
	TFF of F = −0.163 × P of LS+0.191 × P of F+0.168 × P of HP				
TFF of L	VIP	**1.452**	0.515	**1.118**	0.613
	SRC	−0.279	0.099	0.215	0.118
	TFF of L = −0.279 × P of LS+0.215 × P of F				
TFF of S	VIP	0.552	**1.022**	**1.513**	0.600
	SRC	−0.089	−0.164	0.243	−0.096
	TFF of S = −0.164 × P of S+0.243 × P of F				

*APS, Average Pupil Size; TFF, Time to First Fixation; TFD, Total Fixation Duration; P, Proportion; LS, Landscape Space Element; S, Sky Element; F, Facade Element; HP, Hard Paving Element; VIP, Variable Importance in Projection; SRC, Standardized Regression Coefficient; wThe bold value in VIP line is >0.8; 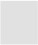, The coefficient used in regression equation*.

#### Correlation Between the Average Distance From the Geometrical Center Point of Space Elements to the Center Point of the Picture and Eye Movement Measures

Through Spearman's rank correlation, the average distance between the GCP of sky element (r = −0.470^*^, *p* = 0.036), facade element (r = −0.594^**^, *p* = 0.006) and landscape space element (r = −0.490^**^, *p* = 0.028) to the CPP were found to be negatively correlated with the TFD of staff on the three elements.

Secondly, the average distance between the GCP of facade element to the CPP was negatively correlated with the TFF of staff on itself (r = −0.572^**^, *p* = 0.008) and APS (r = −0.502^*^, *p* = 0.024). Thirdly, the average distance between the GCP of landscape space element to the CPP was positively correlated with the TFD of staff on the facade element (r = 0.693^**^, *p* = 0.001). Finally, the average distance between the GCP of hard paving element to the CPP was positively correlated with the TFF of staff on itself (r = 0.635^**^, *p* = 0.003) and the TFD of staff on the sky element (r = 0.449^*^, *p* = 0.047) (Table 9).

**Table 9:** Correlations coefficient between the average distance from the GCP of each space elements to the CPP and EMM.

			**TFD of sky element**	**TFD of facade element**	**TFD of landscape space element**	**TFF of hard paving element**	**TFF of facade element**	**APS**
Spearman's rho	average distance from the GCP of sky element to the CPP	Correlation coefficient	−0.470^*^	−0.071	0.089	0.115	0.167	0.393
		Sig. (2-tailed)	0.036	0.767	0.710	0.628	0.481	0.087
	average distance from the GCP of facade element to the CPP	Correlation coefficient	0.222	−0.594^**^	0.338	−0.366	−0.572^**^	−0.502^*^
		Sig. (2-tailed)	0.347	0.006	0.145	0.113	0.008	0.024
	average distance from the GCP of landscape space element to the CPP	Correlation coefficient	0.323	0.693^**^	−0.490^*^	0.375	0.312	0.258
		Sig. (2-tailed)	0.164	0.001	0.028	0.103	0.180	0.273
	average distance from the GCP of hard paving element to the CPP	Correlation coefficient	0.635^**^	0.283	−0.256	0.449^*^	0.093	0.183
		Sig. (2-tailed)	0.003	0.227	0.276	0.047	0.697	0.440

* *Correlation is significant at the 0.05 level (2-tailed)*.

** *Correlation is significant at the 0.01 level (2-tailed)*;

*TFD, Total Fixation Duration; TFF, Time to First Fixation; APS, Average Pupil Size; GCP, Geometrical Center Point; CPP, Center Point of the Picture*.

#### Correlation Between Picture Saturation and Brightness and Eye Movement Measures

Through Spearman's rank correlation, the saturation value of the HORS picture was found to be negatively correlated with the TFD of staff on the facade element (r = −0.593^**^, *p* = 0.006) and the TFF of staff on the landscape space element (r = −0.601^**^, *p* = 0.005). At the same time, the brightness value of the HORS picture was positively correlated with the TFD of staff on the sky element (r = 0.541^*^, *p* = 0.014) and negatively correlated with the APS of staff (r = −0.495^*^, *p* = 0.026) (Table 10).

**Table 10:** Correlations coefficient between the saturation & brightness of sample pictures and EMM.

		**TFD of sky element**	**TFD of facade element**	**TFF of landscape space element**	**Average pupil size**
Spearman's rho	Saturation of picture	−0.322	−0.593^**^	−0.601^**^	−0.150
	Sig. (2-tailed)	0.166	0.006	0.005	0.527
	Brightness of picture	0.541*	0.265	0.353	−0.495*
	Sig. (2-tailed)	0.014	0.259	0.127	0.026

** *Correlation is significant at the 0.01 level (2-tailed)*.

* *Correlation is significant at the 0.05 level (2-tailed)*.

*TFD, Total Fixation Duration; TFF, Time to First Fixation*.

### Correlation Between Self-Rating Restoration Scale Score and Space Attributes

Through Spearman's rank correlation, the proportion of sky element in HORS was found to be positively correlated with the scores of staff in the three dimensions of SRRS: emotional (r = 0.700^**^, *p* = 0.001), cognitive (r = 0.631^**^, *p* = 0.003) and behavioral (r = 0.697^**^, *p* = 0.001). Additionally, the average distance between the GCP of sky element to the CPP was also negatively correlated with the scores of staff in the dimension of cognitive (r = −0.478^*^, *p* = 0.033), while the average distance between the GCP of hard paving element to the CPP was positively correlated with the scores of staff in the three dimensions of SRRS: emotional (r = 0.673^**^, *p* = 0.001), cognitive (r = 0.589^**^, *p* = 0.006) and behavioral (r = 0.598^**^, *p* = 0.005) (Table 11).

**Table 11:** Correlations coefficient between the proportion and location of space elements and the score of self-rating restoration scale.

			**Emotional response**	**Cognitive response**	**Behavioral response**
Spearman's rho	proportion of sky element	Correlation coefficient	0.700^**^	0.631^**^	0.697^**^
		Sig. (2-tailed)	0.001	0.003	0.001
	average distance from the GCP of sky element to the CPP	Correlation coefficient	−0.327	−0.478^*^	−0.378
		Sig. (2-tailed)	0.160	0.033	0.100
	average distance from the GCP of hard paving element to the CPP	Correlation coefficient	0.673^**^	0.589^**^	0.598^**^
		Sig. (2-tailed)	0.001	0.006	0.005

** *Correlation is significant at the 0.01 level (2-tailed)*.

* *Correlation is significant at the 0.05 level (2-tailed)*.

## Discussion

### Pay Attention to the Differentiated Needs of Staff

#### Set Up Hospital Outdoor Rest Space Close to the Working Area of the Group of Medical Staff

The findings of the present study reveal that compared with the administrator group, the doctor and nurse group (summarized as the group of medical staff) had longer TFD of the landscape space element and larger APS, and the two forms of EMM may be related to the working mode of the three groups. Such findings could be attributed to people with restorative needs potentially paying more attention to the landscape space element (33). Moreover, staff may show pupil dilation due to the high attention required for work (34).

In reality, the working place of the nurse is relatively fixed, and there are few opportunities to become exposed to HORS. Doctors would have commuting opportunities for outdoor contact due to the needs of outpatient service, ward inspection and consultation. Owing to the requirements of the inspection system, the administrator needs to shuttle frequently in the hospital area, and there are more opportunities for HORS contact.

Therefore, the medical staff group who are indoors for a long time and have less contact with HORS may have strong restorative needs and have a long TFD on the landscape space element. As such, HORS should be set near the working area of the group of medical staff, or outdoor pocket space should be arranged near the staff exit (35). HORS that could be physically accessed (such as by way of balcony or porch) have great perceived recovery potential for medical staff (36).

#### Pay Attention to the Work Pressure of Junior Nurses

The pupil size of male staff was found to be significantly smaller than that of female staff, which supported the findings that the pupil size of females was larger than that of males when viewing a pleasant and neutral picture (37).

**Figure 2:**
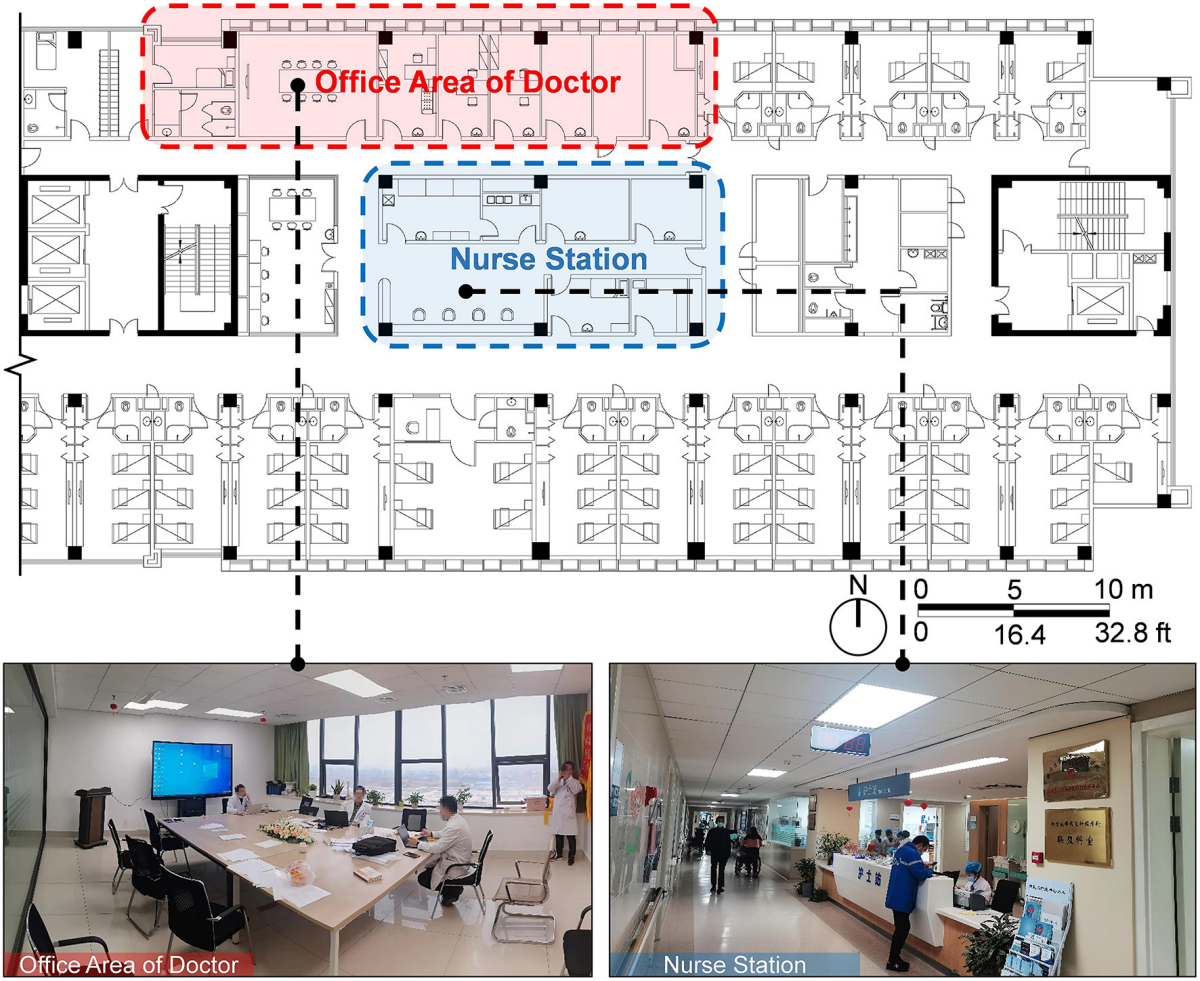
Layout and reality images of the ward where doctors and nurses participated in this study were located.

In medical environment, such findings could be related to the fact that most of the female staff who participated in the study were young nurses, because junior nurses with short working years are more prone to fatigue (38). For the nurses who participated in the present study, compared with the doctor's office area, who may still contact HORS through the window, the centrally arranged nurse station would make it difficult for nurses to contact HORS at work, resulting in more cognitive load and a larger pupil size (Figure 2).

As such, more attention should be given to junior nurses, and a lounge with a view of HORS could be set up around the nurses' station to prevent nurses' fatigue (39). Because observing HORS could not only effectively reduce the fatigue and pressure of nurses (40), but also have a positive impact on the recovery of patients (41). When the space is limited, biological pictures could also be arranged to provide a positive impact, because the cognitive load required by natural scenes is small (28).

#### Understand the Actual Needs of Senior Staff

With the increase of age, the pupil size may gradually decrease (42). However, in the medical environment, the negative correlation between staff age and APS may also be related to the fact that older staff have rich clinical experience, and also due to work pressure decreasing with age and resulting in a smaller pupil size (43).

At the same time, such factors explain the negative correlation between the age and the TFD of staff on the landscape space element. Since the degree of emotional exhaustion of senior staff in the hospital is significantly lower than that of junior staff (44), the relatively low restorative needs of senior staff may also be the reason for the shortening of their TFD on the landscape space element.

Further, the positive correlation between the age and the TFF of staff on the hard artificial elements shows that the older a staff member is, the harder it is to quickly identify and find the hard artificial elements in HORS. Thus, the eye-catching degree of the height difference of hard artificial elements in HORS needs to be improved through reflective strips or color identification, so as to prevent senior staff from falling due to their failure to find the height difference in time.

### Influence of Spatial Attributes on Eye Movement Measures of Staff

#### The Positive Effects of the Natural Environment and the Negative Effects of Hard Artificial Elements

The results show that the proportion of various elements in HORS pictures is a significant factor that affects the EMM of staff.

Firstly, from the influence of the proportion of space element on pupil size, a smaller pupil size indicates a lower cognitive load (43). Therefore, the sky element would not only reduce the pressure of staff and produce a restorative effect (45), but also result in the pupil size contraction of staff. Such effects could be ascribed to the open scene represented by the sky element being more likely to reduce pressure (28), while the urban scene represented by the hard paving element would lead to a higher cognitive load (46).

Secondly, in terms of the proportion of space elements and the TFD of staff on each element people would pay more attention to the landscape space element because the natural scene is more restorative compared with the urban environment (47, 48). Therefore, the EMM results effectuated by a higher proportion of landscape space element would also bring benefits to staff and also help to reduce medical errors (49).

Conversely, a higher proportion of hard artificial elements would not help staff recover because the TFD of staff on itself would be increased. Additionally, the TFD of staff on the sky element was affected by the proportion of landscape space element, which shows that staff may pay more attention to landscapes with more changes in tone and color (50). The edge space of the facade also had a strong visual attraction for people (51), and thus, the sky element in contact with the edge of the facade element would also get more attention from staff.

Finally, in terms of the proportion of elements and the TFF of staff on each element, a higher proportion of landscape space element would help to shorten the TFF of staff on and allow for recovery from the short contact (52), since people like landscapes in sharp contrast to artificial elements (53). Because staffs have little time to use HORS or only regarded such spaces as passages (54), therefore, improving the visibility of landscape space element in HORS is of positive significance for its rapid discovery by staffs.

In addition, due to the high proportion of natural environment in HORS, hard artificial elements would also be easily identified (55), which shortens the TFF of staff. However, it should also be noted that the high proportion of hard artificial elements would also divide the attraction of landscape (24), because people prefer moderate artificial intervention in a restorative environment (56).

#### Positive Impact of Place the Natural Environment in the Visual Center

The correlation results demonstrate that the position of space elements in the HORS pictures is a significant influencing factor for the EMM of staff. Firstly, for the farther distance between the GCP of the sky and landscape space elements to the CPP, the staff would increase the TFD on the facade element while decreasing the TFD on the sky and landscape space elements. Here, the possible observation perspective of staff should be understood in the HORS layout, and the natural environment should be arranged in the visual center to increase the TFD of staff on the sky and landscape space elements. Meanwhile, the TFD of staff on the facade element should be reduced to produce a restorative effect.

Secondly, the facade element away from the visual center may not only reduce the TFD of staff on itself but also contribute to the contraction of pupil size due to the increased sense of opening caused by the decentralized layout of the facade element (23). At the same time, the correlation between the distance between the GCP of the facade element to the CPP and the TFF of staff on the facade element is further evidence of the aforementioned results that the facade element at the edge of the picture may be highlighted by the “background” composed of green plants.

Finally, the farther distance between the GCP of the hard paving element to the CPP, the TFD of staff on the sky element may be further increased, while helping to reduce the eye-catching degree and improve the scores of staff in the SRRS. Hence, in the hard paving element design of HORS, a non-centered and sinuous layout pattern should be considered, so as to stimulate the pathfinding behavior of staff, weaken the eye-catching degree of the hard paving element and the TFD of staff, and achieve a better self-rating restoration result.

#### Positive Impact of Improving the Brightness and Saturation of Space

The positive significance of high saturation in HORS was observed in the present study. Therefore, in the actual design, more colored items such as flowers could be planted to attract staff, allowing them to quickly identify and discover the landscape space element while reducing their attention to the facade element.

At the same time, higher brightness in HORS was found to be conducive to the contraction of pupil size while increasing the TFD of staff to the sky element, that is, higher brightness in HORS had a better restorative value and positive effect (57). In addition, the influence of saturation and brightness found was also further evidence of the relevant research results (58).

### Provide Staff With Access to the Sky Environment in a Variety of Ways

In terms of spatial attributes and psychological evaluation, a high proportion of sky element was found to be helpful for improving the staff SRRS scores. In addition, the sky element arranged in the visual center also helped to improve the staff SRRS score at the cognitive level.

Such findings are further evidence of the negative influence of the proportion of sky element on pupil size. Because medical staff rarely have the opportunity to contact with the outdoor environment, providing them with a HORS with more sky element would produce a better restorative effect, and the sky element could activate the areas related to space cognition and behavioral perception in people's brains and produce beneficial effects (59).

Thus, in the actual design, providing staff with access to the sky environment in a variety of ways could be considered. To be specific, while increasing the proportion of the sky element in HORS from the perspective of direct physical access, and also providing a sky environment with direct visual access for staffs who cannot get away from work, such as in the ward of higher floors, a larger scale sky view could be provided by means of windows (59).

## Conclusion

**Figure 3:**
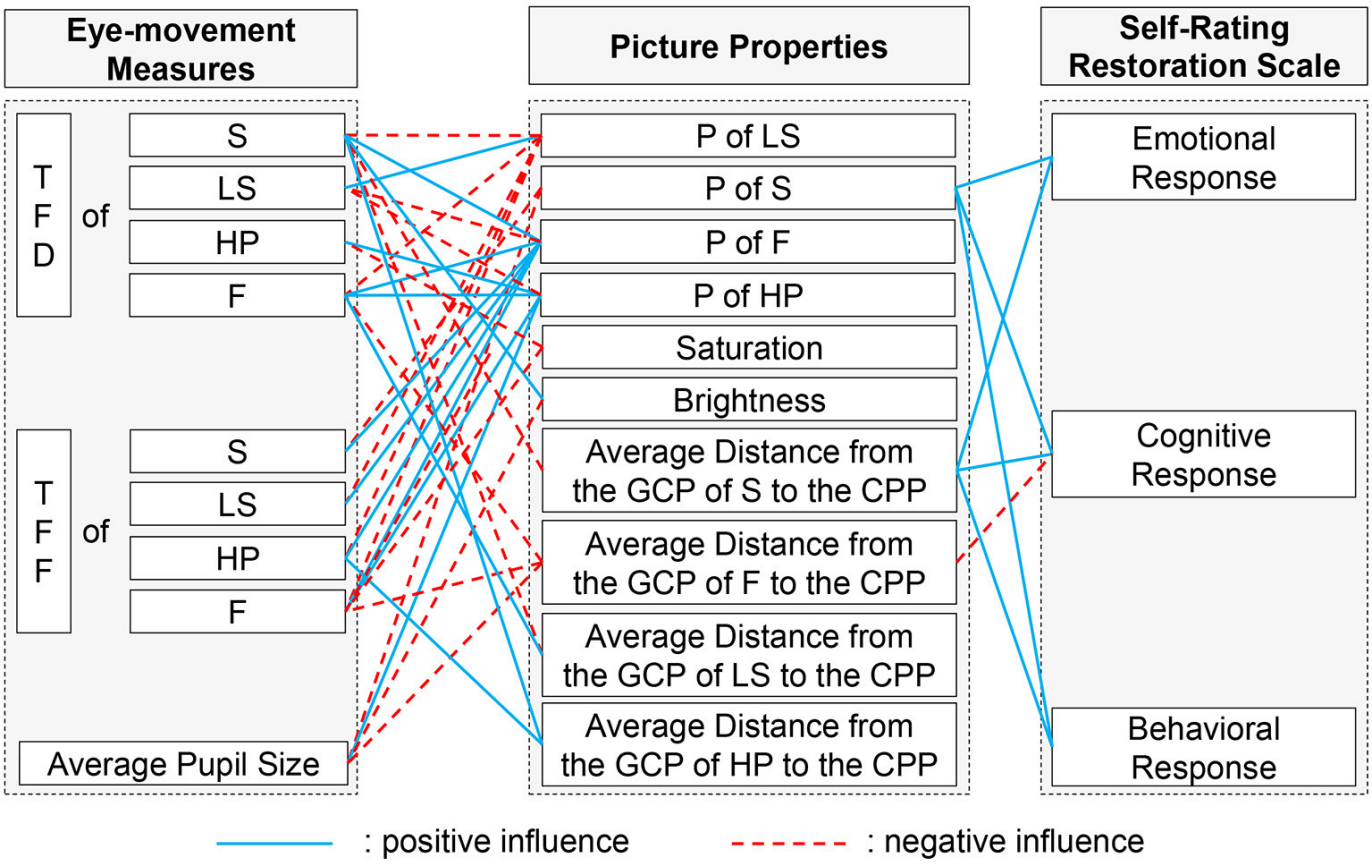
Relationship between the proportion of picture properties and EMM and SRRS score. TFD, Total Fixation Duration; TFF, Time to First Fixation; P, Proportion; S, Sky Element; F, Facade Element; LS, Landscape Space Element; HP, Hard Paving Element; GCP, Geometrical Center Point; CPP, Center Point of the Picture.

With the focus on the impact of HORS on staffs' recovery, this study investigated the effect of HORS on the EMM and self-rating restoration of staff. The relationships between HORS picture attributes and the EMM and SRRS of staff were summarized (Figure 3).

On this basis, through further analysis, the research puts forward the following specific suggestions for the optimization practice of HORS. (1) The outdoor rest space should be set up close to the working area of the group of medical staff. (2) Enhance the visibility of the height difference of hard artificial elements in HORS. (3) A lounge with a view of HORS could be set up around the nurses' station. (4) The proportion of natural environment, saturation, and brightness of HORS should be increased, and the proportion of hard artificial elements should be reduced. (5) The natural environment should be placed in the visual center of HORS; (6) The proportion of the sky element in HORS should be increased, or introduce the sky into the indoor environment through windows.

In summary, the present study is not only an innovation in method selection and object selection but also a practical verification of evidence-based design in Chinese HORS design, which provides enlightenment for the promotion and application of evidence-based design in T1GHs in China.

Besides, only Chinese T1GHs were selected as the research object, and there was a lack of attention paid to specialized hospitals such as children, rehabilitation, and orthopedics. Because different types of hospitals may have differences in HORS and the restorative needs of staff. Secondly, to avoid complex situations, the experimental environment and tasks were controlled and simplified, to not interact with the real HORS scene and there may be a gap with the actual environment. Thus, in future research, hospital staff could be invited to wear head-mounted eye tracker devices or with the help of virtual reality technology to explore the actual environment. Thirdly, the influence of weather conditions and a person's mood could be further researched, to identify the impact mechanism. Finally, based on mastering the EMM of staff, the EMM of patient groups could be further explored, fit the needs of the two groups, and build a complete HORS design proposal.

## Data Availability Statement

The raw data supporting the conclusions of this article will be made available by the authors, without undue reservation.

## Ethics Statement

The studies involving human participants were reviewed and approved by the Medical Ethics Committee of the Second Affiliated Hospital of Bengbu Medical College. Written informed consent for participation was not required for this study in accordance with the national legislation and the institutional requirements. Written informed consent was obtained from the individual(s) for the publication of any potentially identifiable images or data included in this article.

## Author Contributions

All authors listed have made a substantial, direct, and intellectual contribution to the work and approved it for publication.

## Funding

This research was funded by the Ministry of Education of the People's Republic of China Project of Humanities and Social Sciences (Grant Nos. 17YJAZH047 and 20YJC760119) and National Natural Science Foundation of China (Grant No. 52178012) and the Fundamental Research Funds for the Central Universities (Grant No. PA2021KCPY0038).

## Conflict of Interest

The authors declare that the research was conducted in the absence of any commercial or financial relationships that could be construed as a potential conflict of interest.

## Publisher's Note

All claims expressed in this article are solely those of the authors and do not necessarily represent those of their affiliated organizations, or those of the publisher, the editors and the reviewers. Any product that may be evaluated in this article, or claim that may be made by its manufacturer, is not guaranteed or endorsed by the publisher.
