# Supplementary material for: Influence of Hospital Outdoor Rest Space on the Eye Movement Measures and Self-Rating Restoration of Staff
Source: Front Public Health. 2022 Mar 16;10:855857. doi: 10.3389/fpubh.2022.855857 (PMC8965843; doi:10.3389/fpubh.2022.855857)
Supplement: Supplementary file 1 [file Data_Sheet_1.PDF]

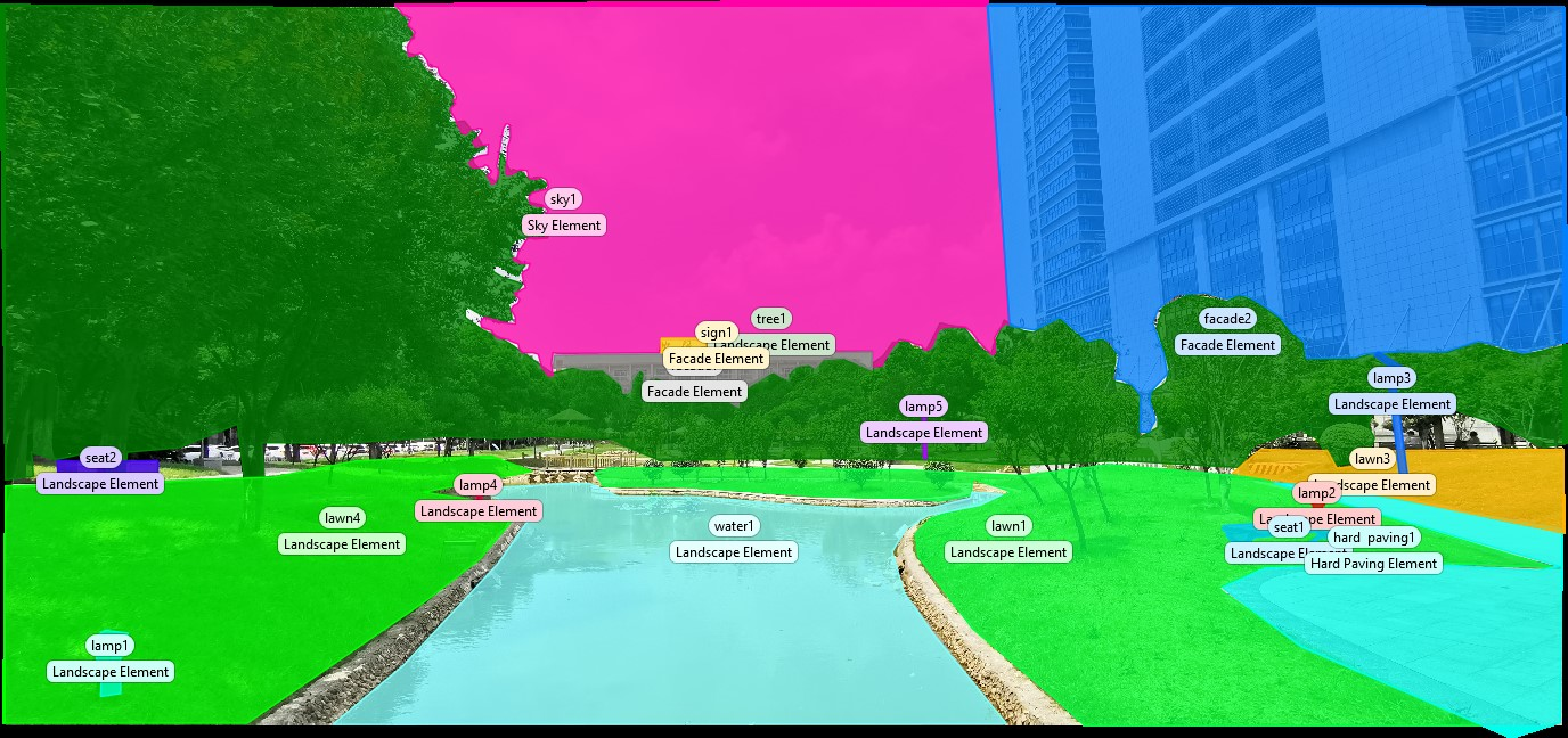

sky1
Sky Element
tree1
sign1
Facade Element
Facade Element
facade2
Facade Element
lamp3
Landscape Element
lamp5
Landscape Element
seat2
Landscape Element
lamp4
Landscape Element
lawn4
Landscape Element
water1
Landscape Element
lawn1
Landscape Element
lawn3
lamp2
seat1
hard paving1
Hard Paving Element
lamp1
Landscape Element

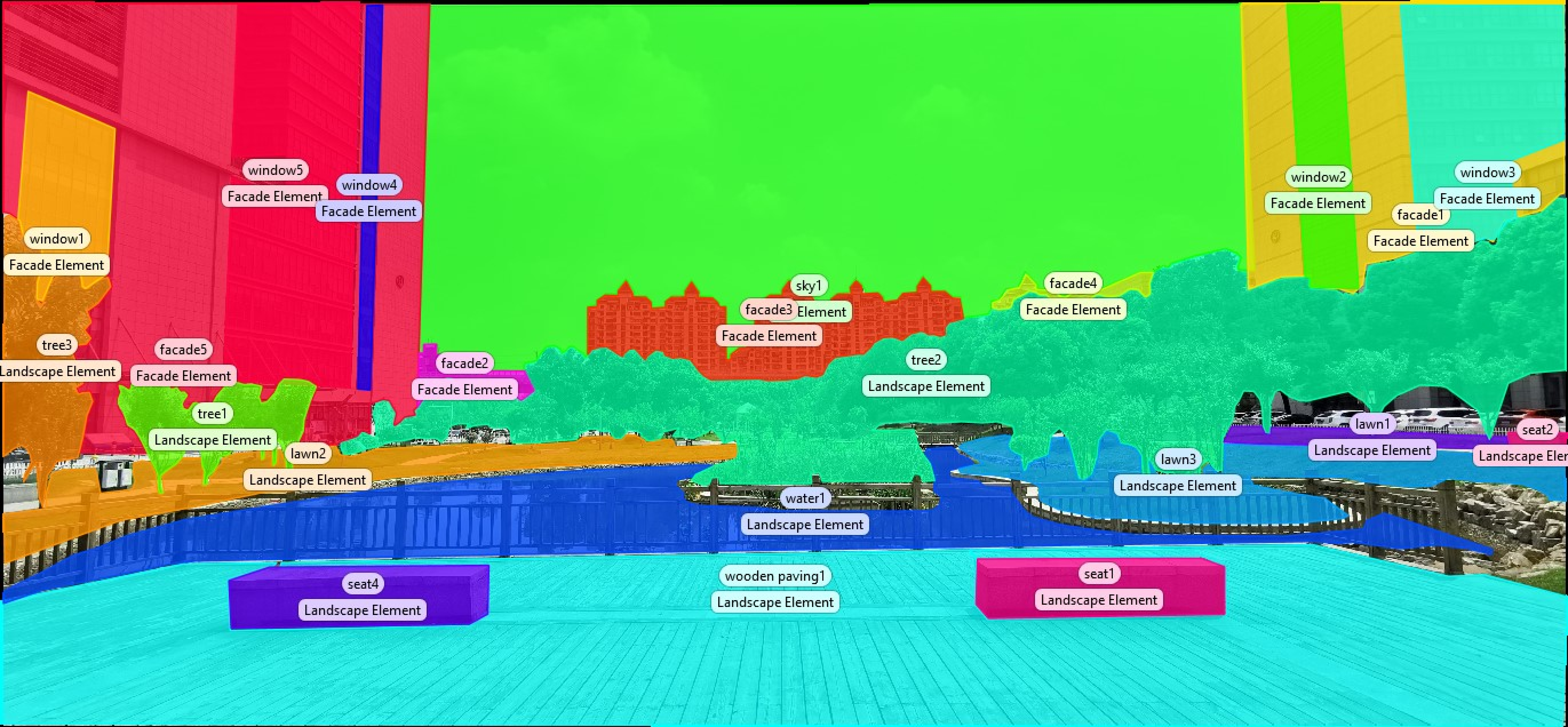
window5
Facade Element
window4
Facade Element
window1
Facade Element
tree3
Landscape Element
facade5
Facade Element
tree1
Landscape Element
lawn2
Landscape Element
facade2
Facade Element
sky1
Element
facade3
Facade Element
tree2
Landscape Element
facade4
Facade Element
window2
Facade Element
window3
Facade Element
facade1
Facade Element
lawn1
Landscape Element
seat2
lawn3
Landscape Element
water1
Landscape Element
wooden paving1
Landscape Element
seat4
Landscape Element
seat1
Landscape Element

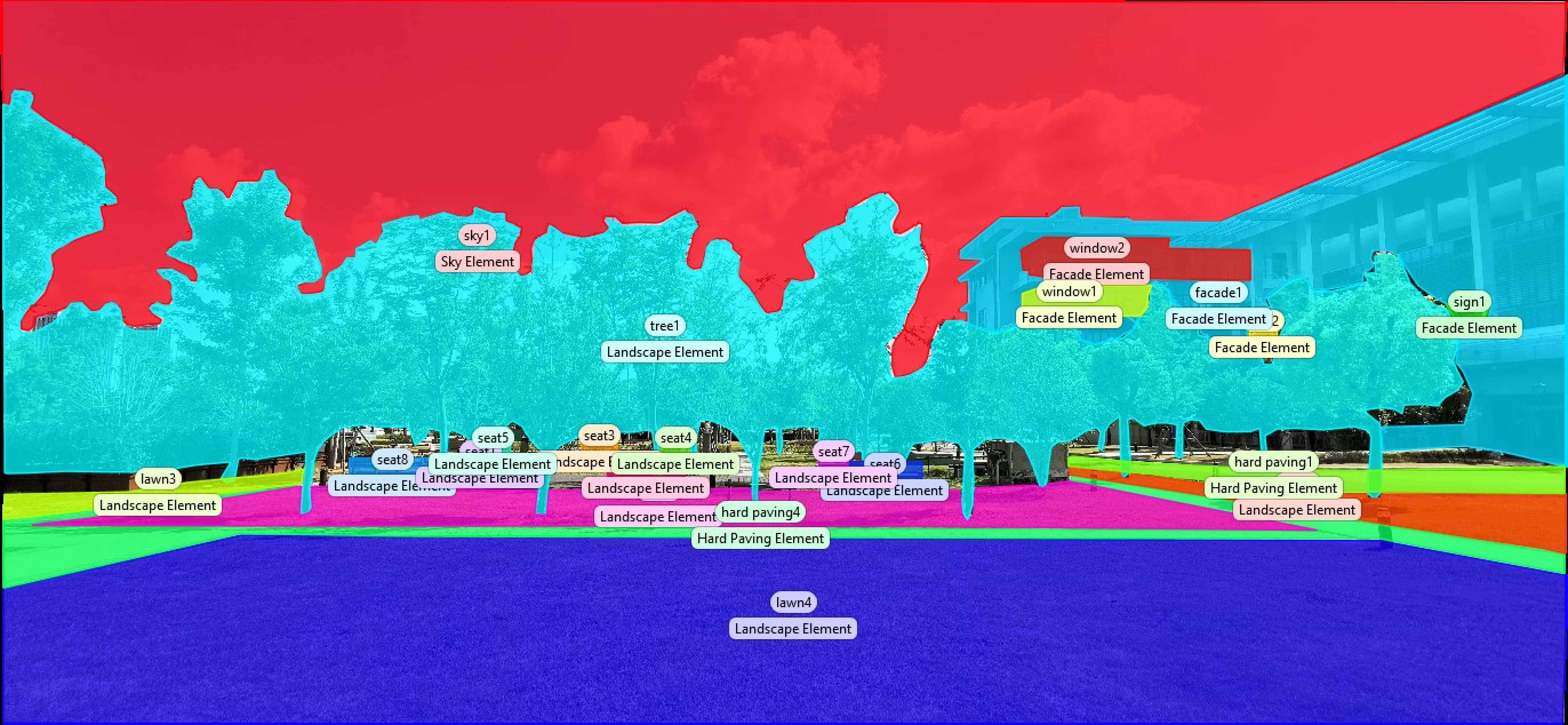
sky1
Sky Element
window2
Facade Element
window1
Facade Element
facade1
Facade Element
Facade Element
sign1
Facade Element
tree1
Landscape Element
seat5
Landscape Element
seat3
seat4
Landscape Element
seat8
seat7
Landscape Element
Landscape Element
lawn3
Landscape Element
Landscape Element
hard paving4
Hard Paving Element
hard paving1
Hard Paving Element
Landscape Element
lawn4
Landscape Element

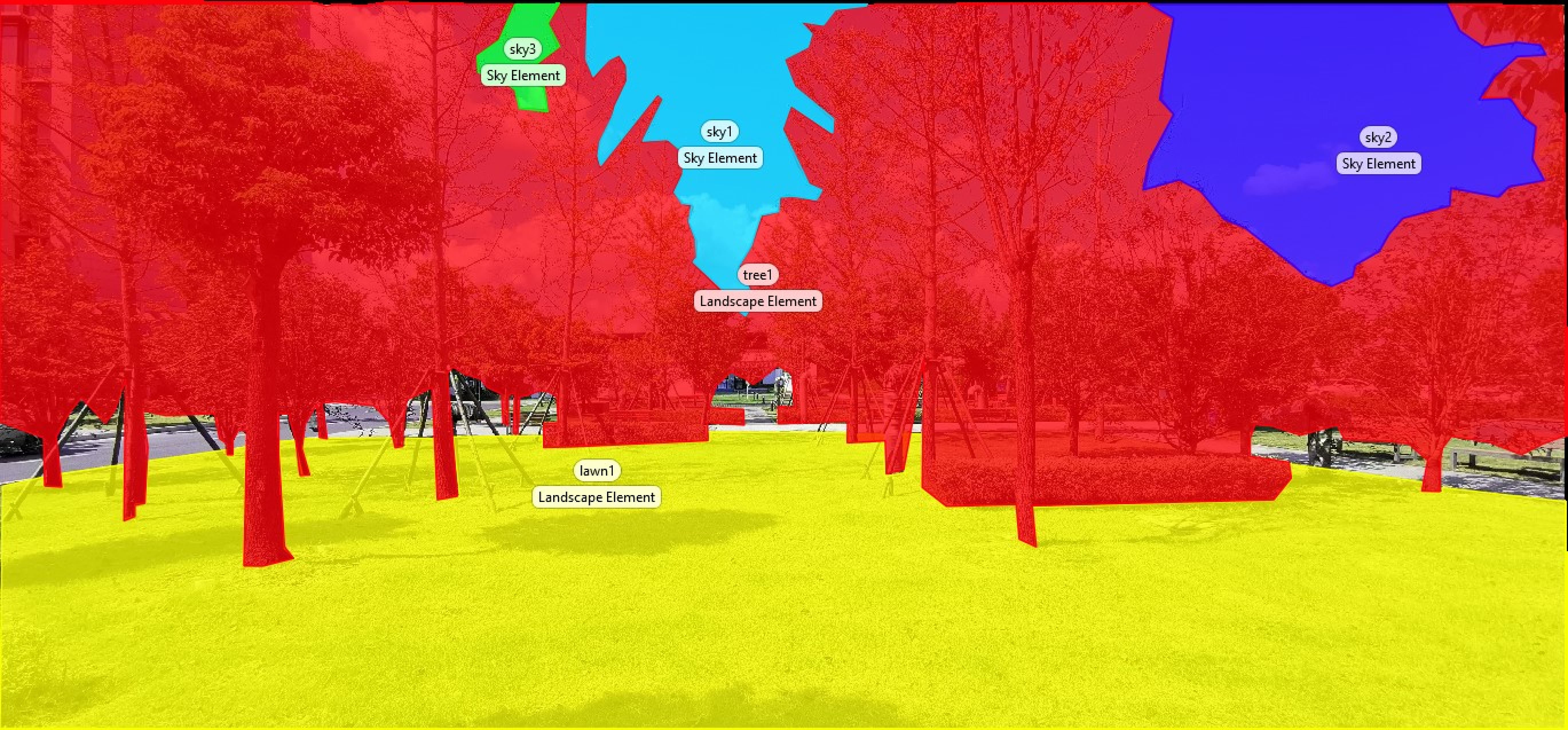
sky3
Sky Element
sky1
Sky Element
sky2
Sky Element
tree1
Landscape Element
lawn1
Landscape Element

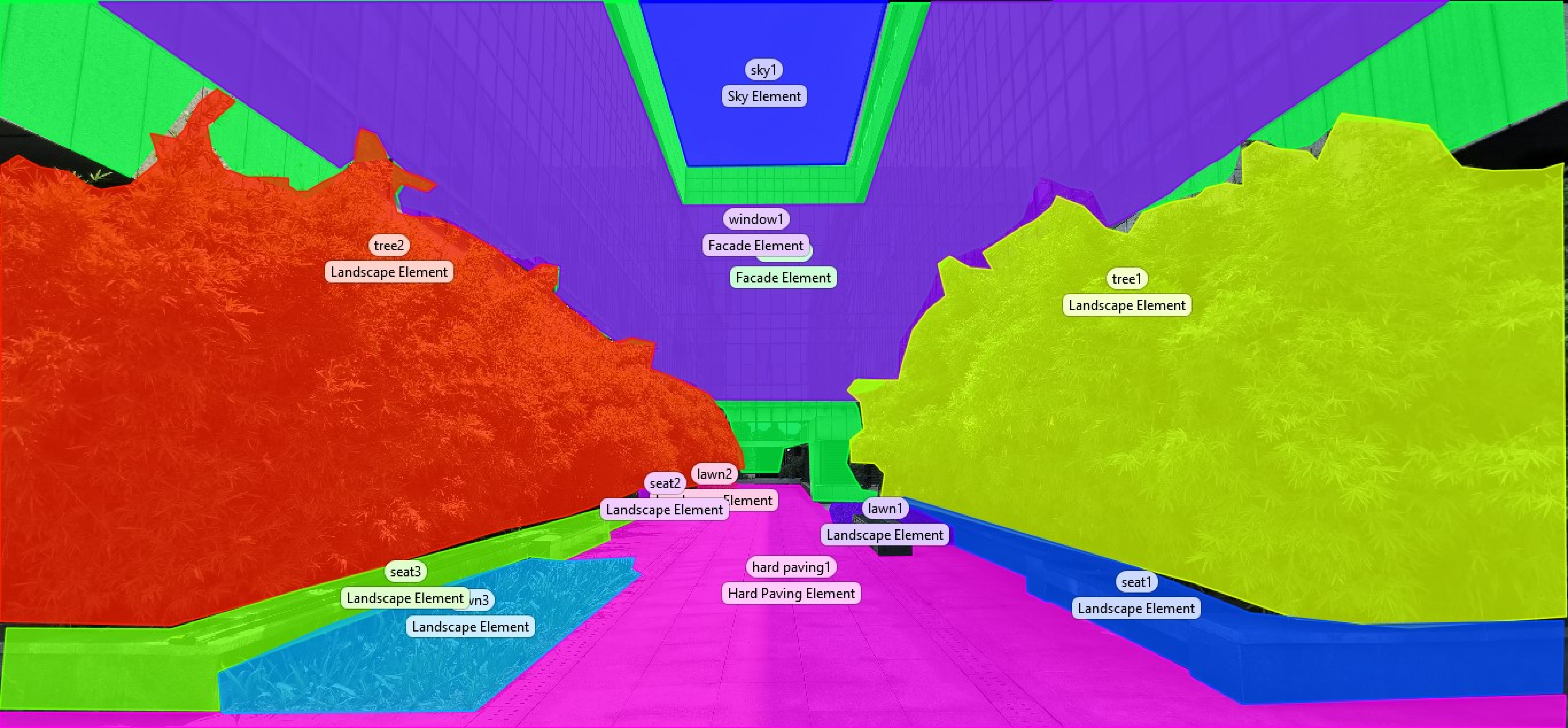
sky1
Sky Element
window1
Facade Element
Facade Element
tree2
Landscape Element
tree1
Landscape Element
lawn2
seat2
Landscape Element
lawn1
Landscape Element
hard paving1
Hard Paving Element
seat3
Landscape Element
Landscape Element
seat1
Landscape Element

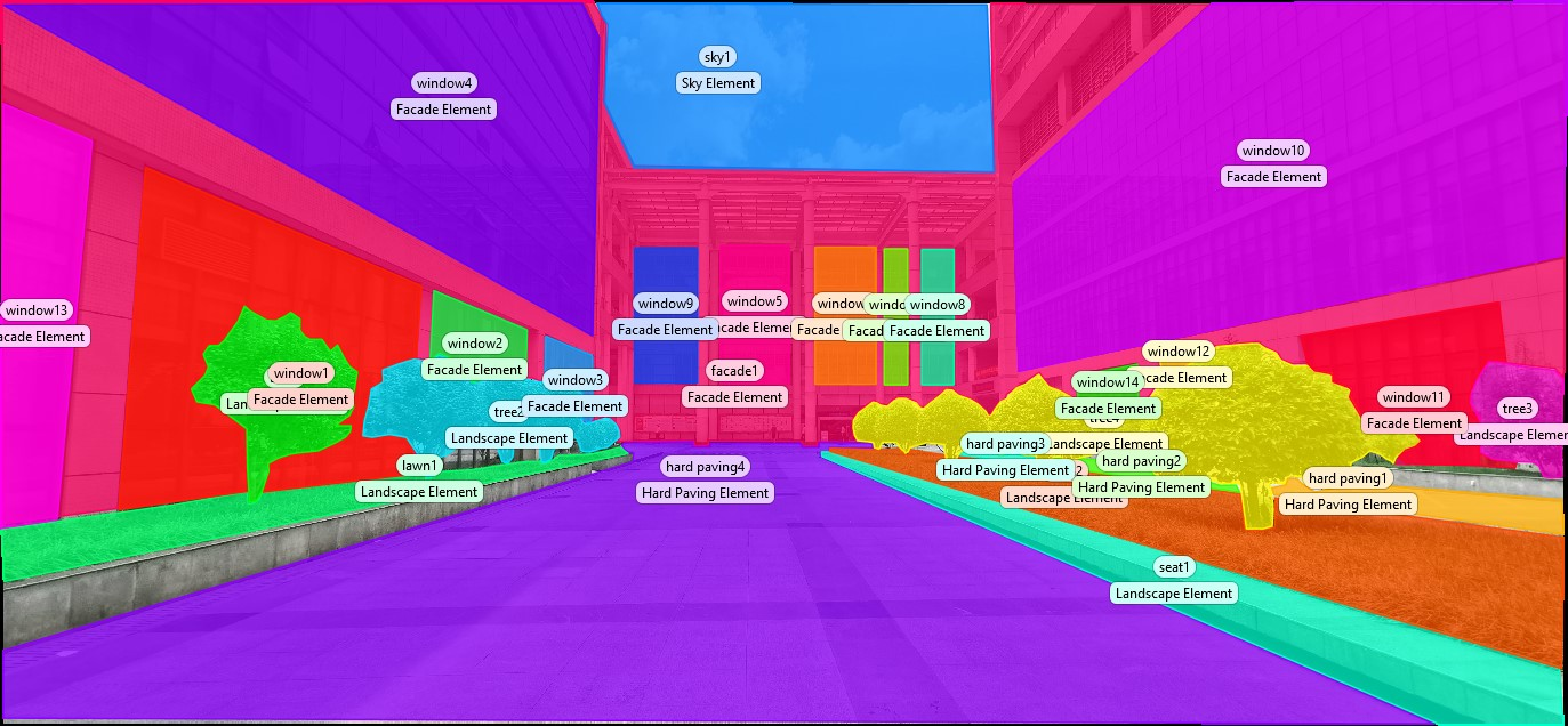
sky1
Sky Element
window4
Facade Element
window10
Facade Element
window13
window9
Facade Element
window5
window8
Facade Element
window2
Facade Element
window1
Facade Element
window3
Facade Element
facade1
Facade Element
window12
window14
Facade Element
window11
Facade Element
tree2
tree3
Landscape Element
hard paving4
Hard Paving Element
lawn1
Landscape Element
hard paving3
Hard Paving Element
hard paving2
Hard Paving Element
hard paving1
Hard Paving Element
seat1
Landscape Element

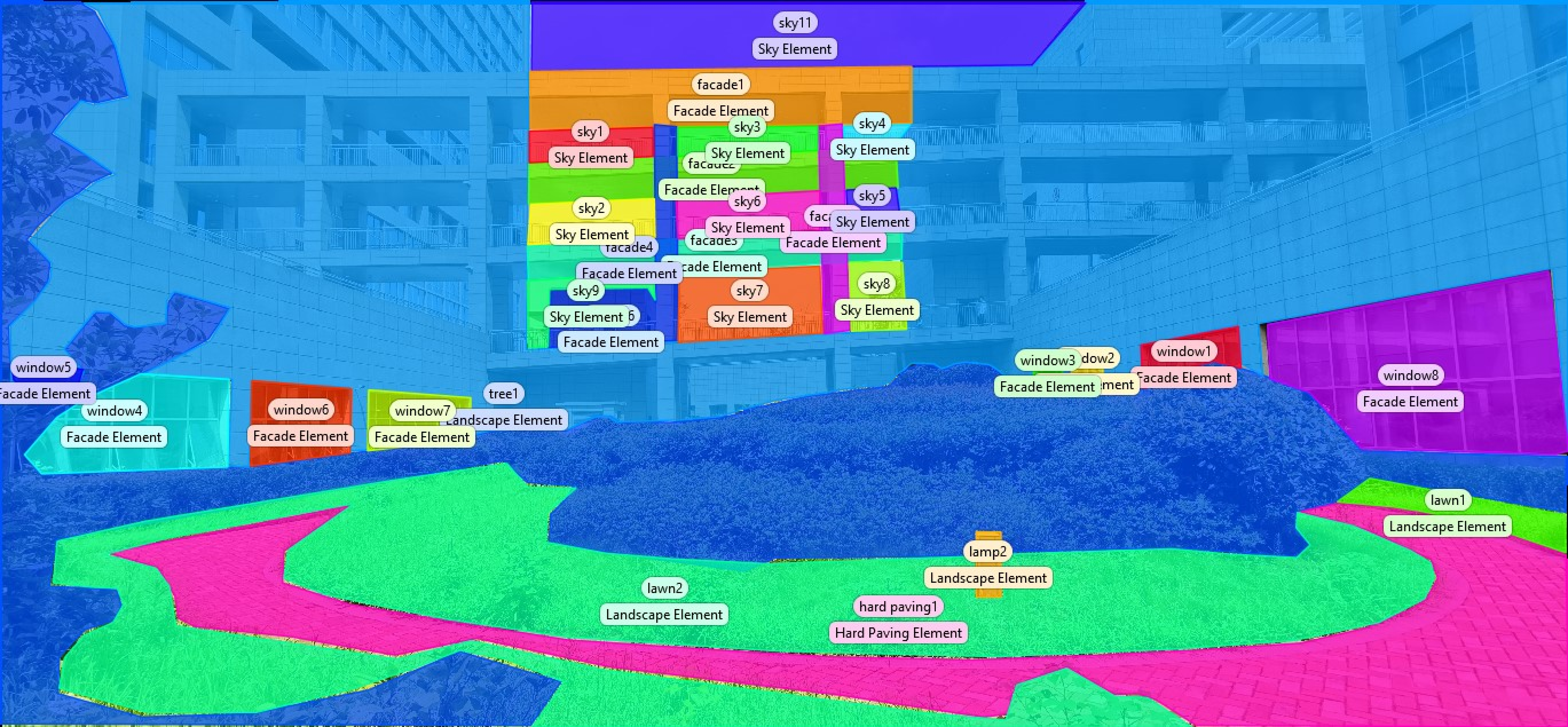
sky11
Sky Element
facade1
Facade Element
sky1
Sky Element
sky3
Sky Element
sky4
Sky Element
Facade Element
sky6
sky5
Sky Element
sky2
Sky Element
Sky Element
Facade Element
Facade Element
sky9
Sky Element
sky7
Sky Element
sky8
Sky Element
Facade Element
window3
window1
Facade Element
Facade Element
window8
Facade Element
window5
window4
Facade Element
window6
Facade Element
window7
Facade Element
tree1
lawn1
Landscape Element
lamp2
Landscape Element
lawn2
Landscape Element
hard paving1
Hard Paving Element

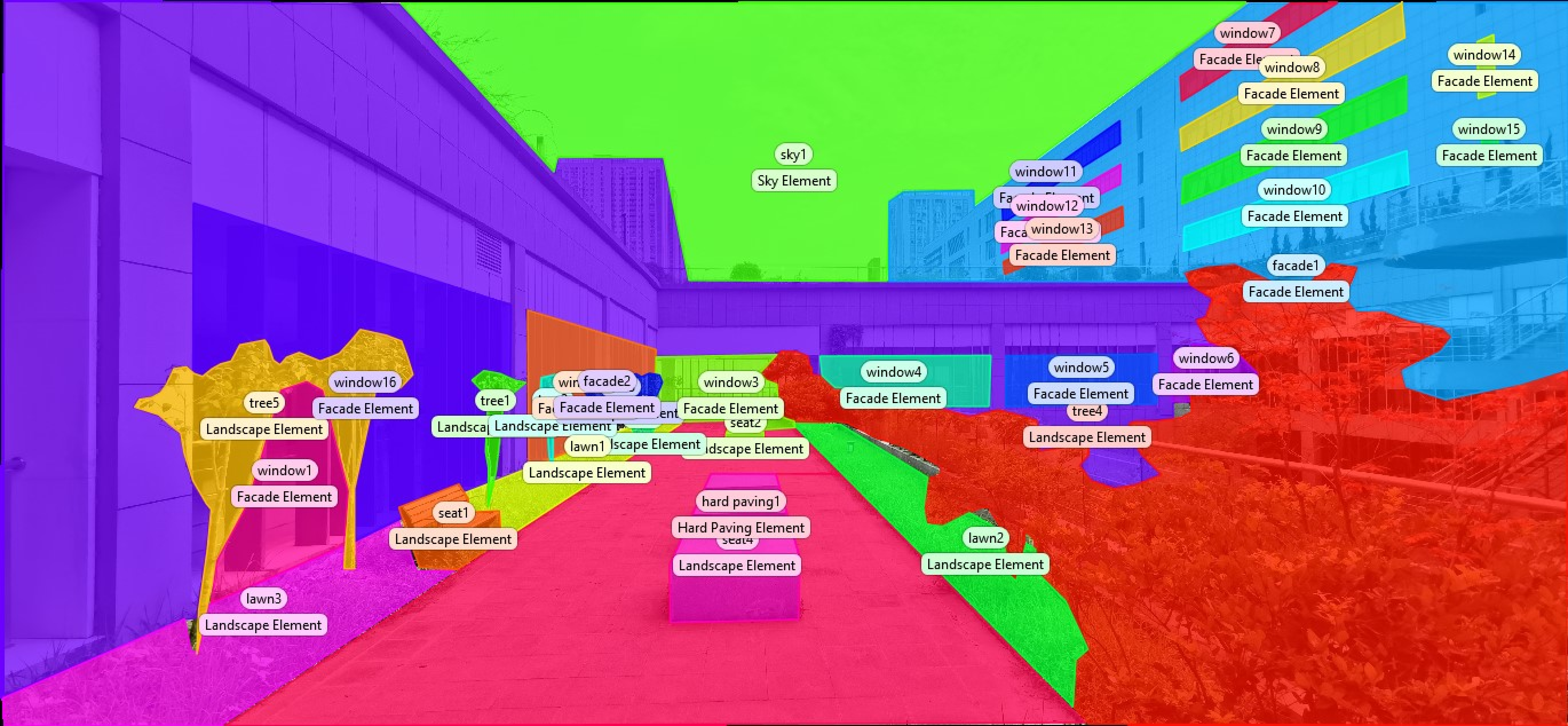
window7
window8
Facade Element
window14
Facade Element
sky1
Sky Element
window9
Facade Element
window15
Facade Element
window11
window12
window13
Facade Element
window10
Facade Element
facade1
Facade Element
window4
Facade Element
window5
Facade Element
window6
Facade Element
tree4
Landscape Element
window16
Facade Element
tree5
Landscape Element
tree1
facade2
Facade Element
window3
Facade Element
lawn1
Landscape Element
window1
Facade Element
seat1
Landscape Element
hard paving1
Hard Paving Element
Landscape Element
lawn2
Landscape Element
lawn3
Landscape Element

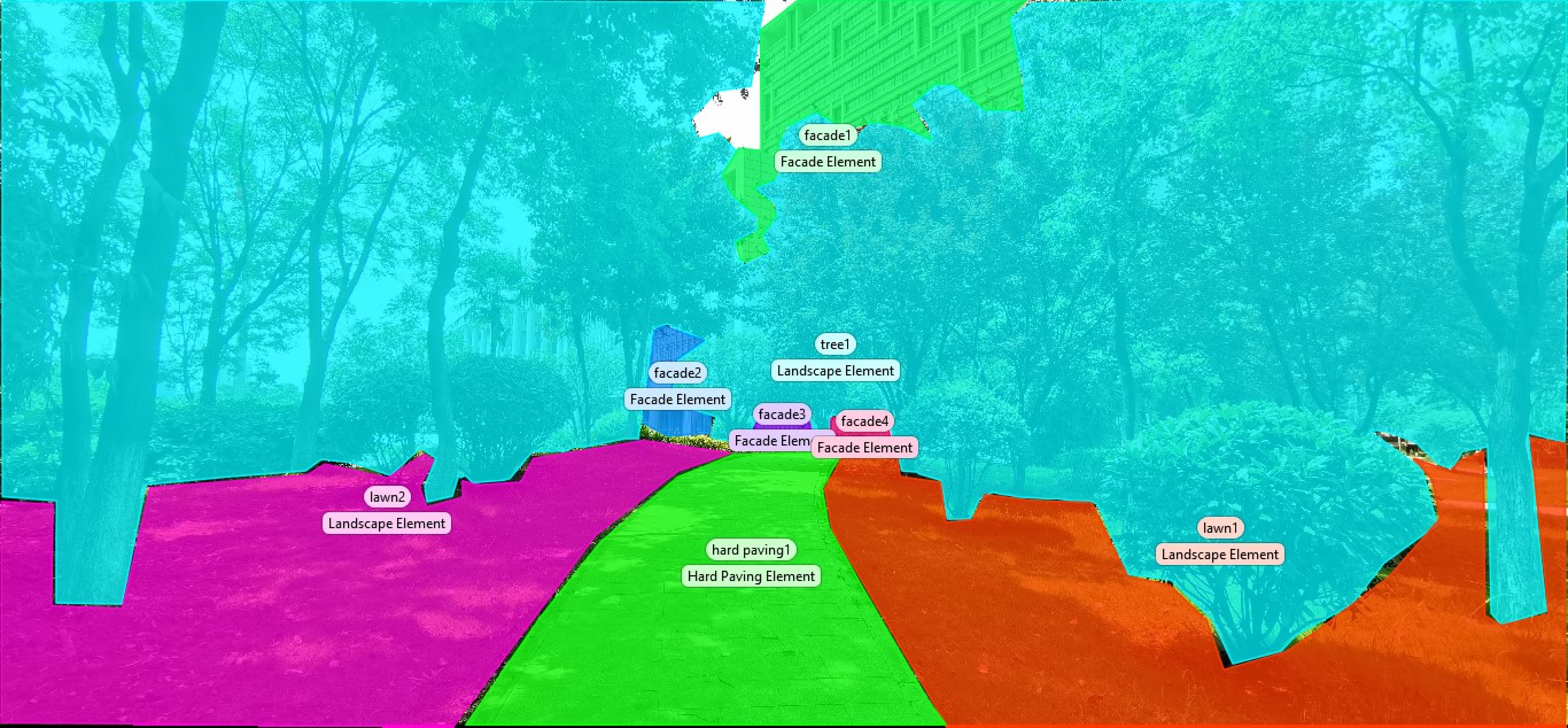
facade1
Facade Element
facade2
Facade Element
tree1
Landscape Element
facade3
Facade Elem
facade4
Facade Element
lawn2
Landscape Element
hard paving1
Hard Paving Element
lawn1
Landscape Element

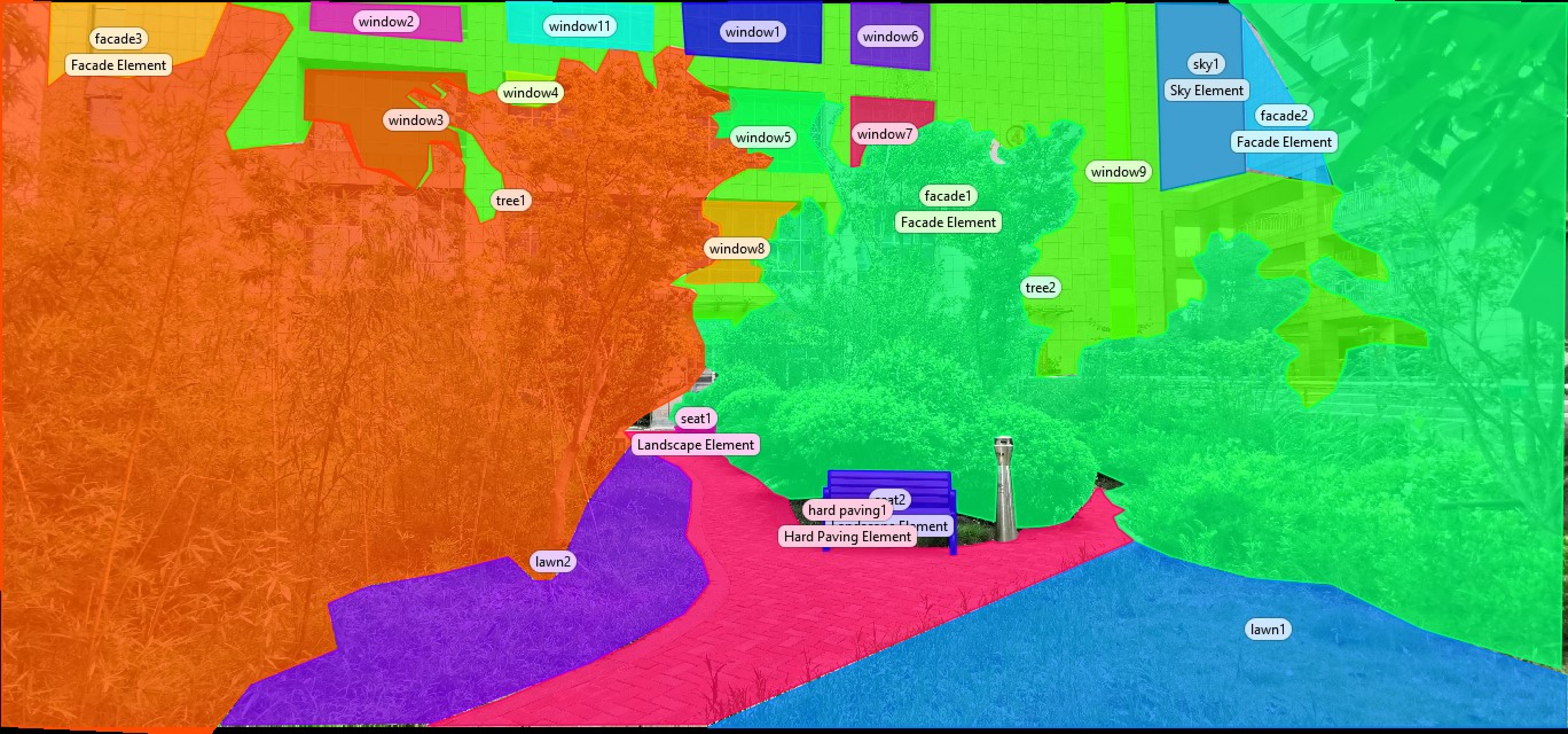
facade3
Facade Element
window2
window11
window1
window6
sky1
Sky Element
facade2
Facade Element
window4
window3
window5
window7
window9
tree1
facade1
Facade Element
window8
tree2
seat1
Landscape Element
hard paving1
Hard Paving Element
lawn2
lawn1

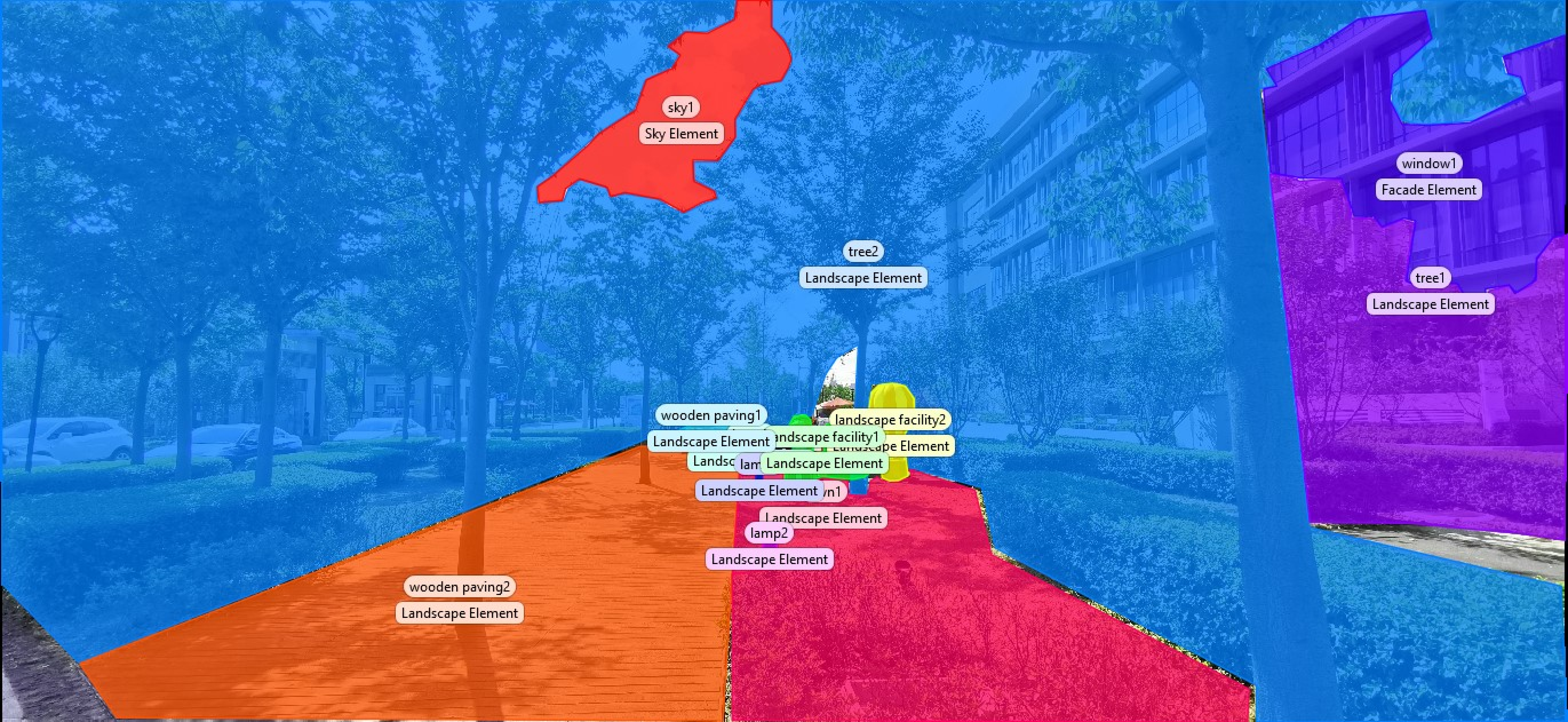
sky1
Sky Element
window1
Facade Element
tree2
Landscape Element
tree1
Landscape Element
wooden paving1
Landscape Element
landscape facility2
Landscape Element
Landscape Element
lamp2
Landscape Element
wooden paving2
Landscape Element

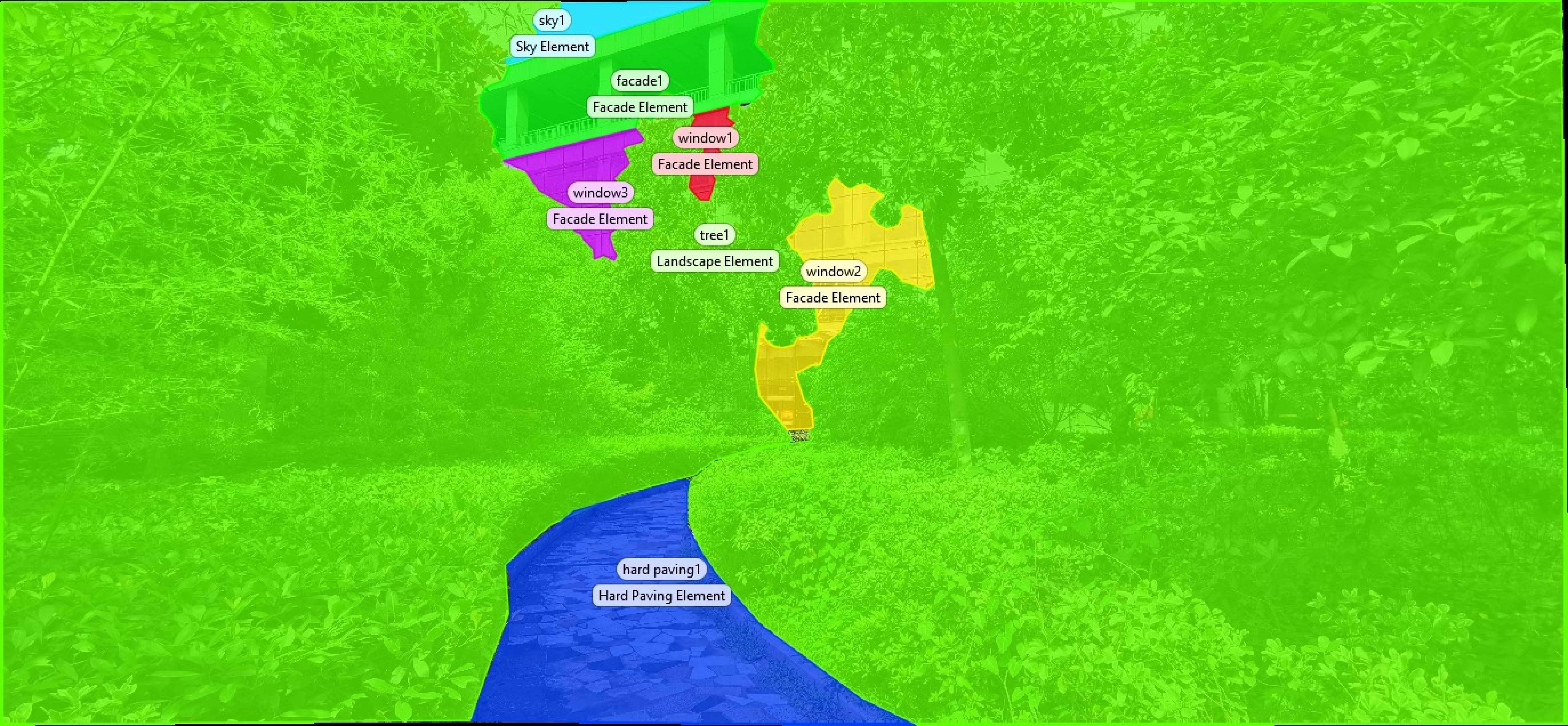

sky1
Sky Element
facade1
Facade Element
window1
Facade Element
window3
Facade Element
tree1
Landscape Element
window2
Facade Element
hard paving1
Hard Paving Element

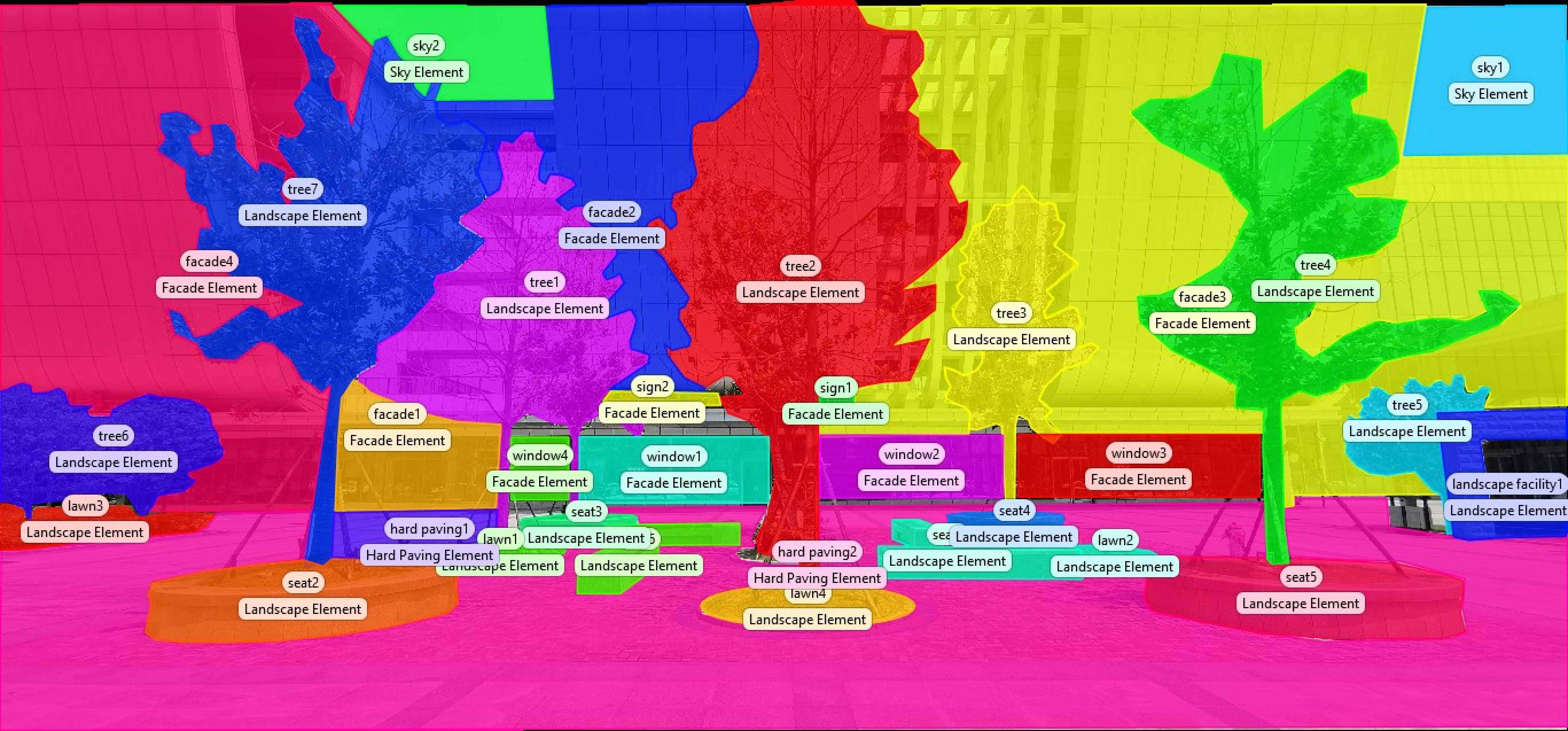
sky2
Sky Element
sky1
Sky Element
tree7
Landscape Element
facade2
Facade Element
facade4
Facade Element
tree1
Landscape Element
tree2
Landscape Element
tree4
Landscape Element
facade3
Facade Element
tree3
Landscape Element
sign2
Facade Element
sign1
Facade Element
facade1
Facade Element
tree5
Landscape Element
tree6
Landscape Element
window4
Facade Element
window1
Facade Element
window2
Facade Element
window3
Facade Element
landscape facility1
Landscape Element
lawn3
Landscape Element
seat3
Landscape Element
seat4
Landscape Element
hard paving1
Hard Paving Element
lawn1
Landscape Element
Landscape Element
Landscape Element
lawn2
Landscape Element
hard paving2
Hard Paving Element
seat2
Landscape Element
lawn4
Landscape Element
seat5
Landscape Element

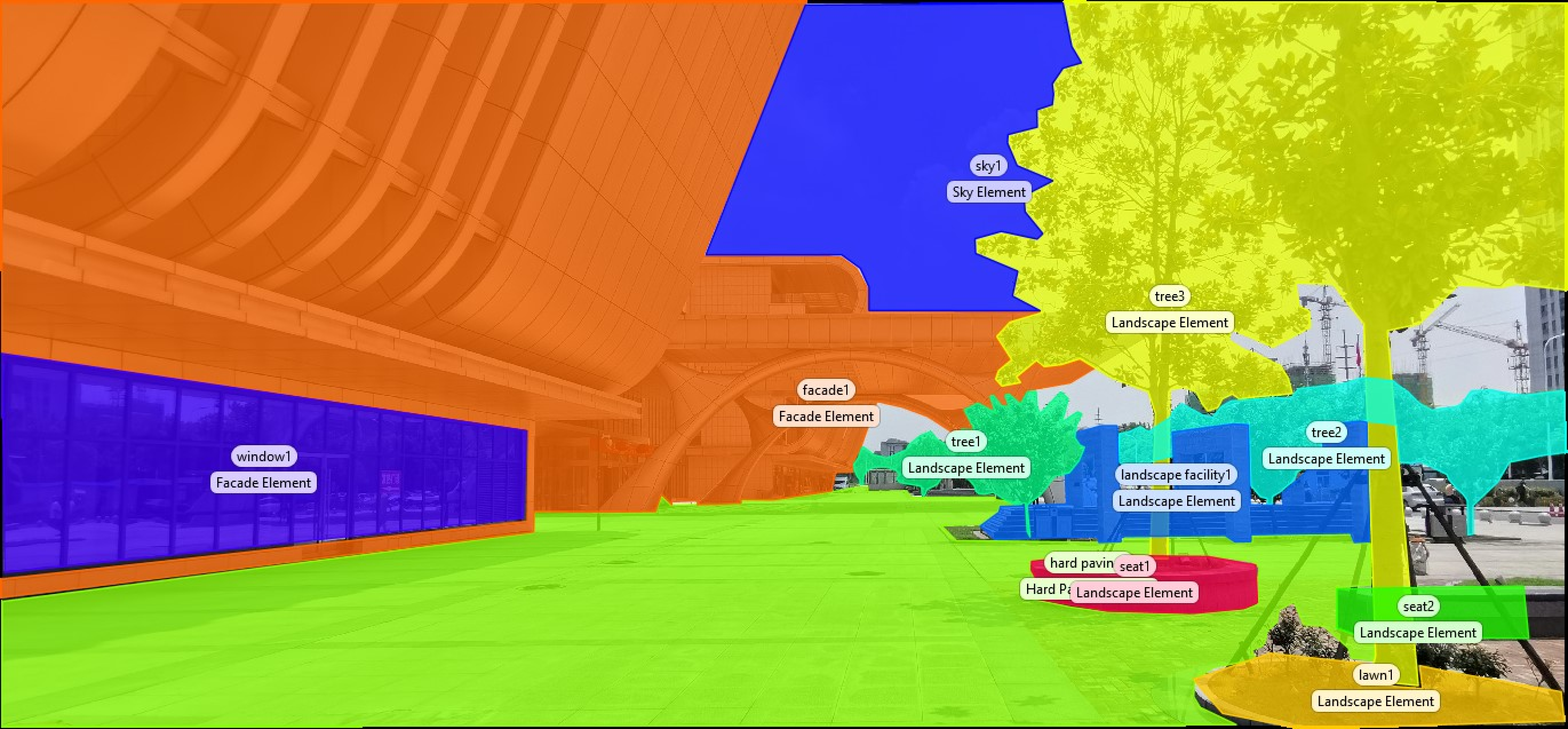
sky1
Sky Element
tree3
Landscape Element
facade1
Facade Element
window1
Facade Element
tree1
Landscape Element
landscape facility1
Landscape Element
tree2
Landscape Element
hard pavin
seat1
Hard Pa
Landscape Element
seat2
Landscape Element
lawn1
Landscape Element

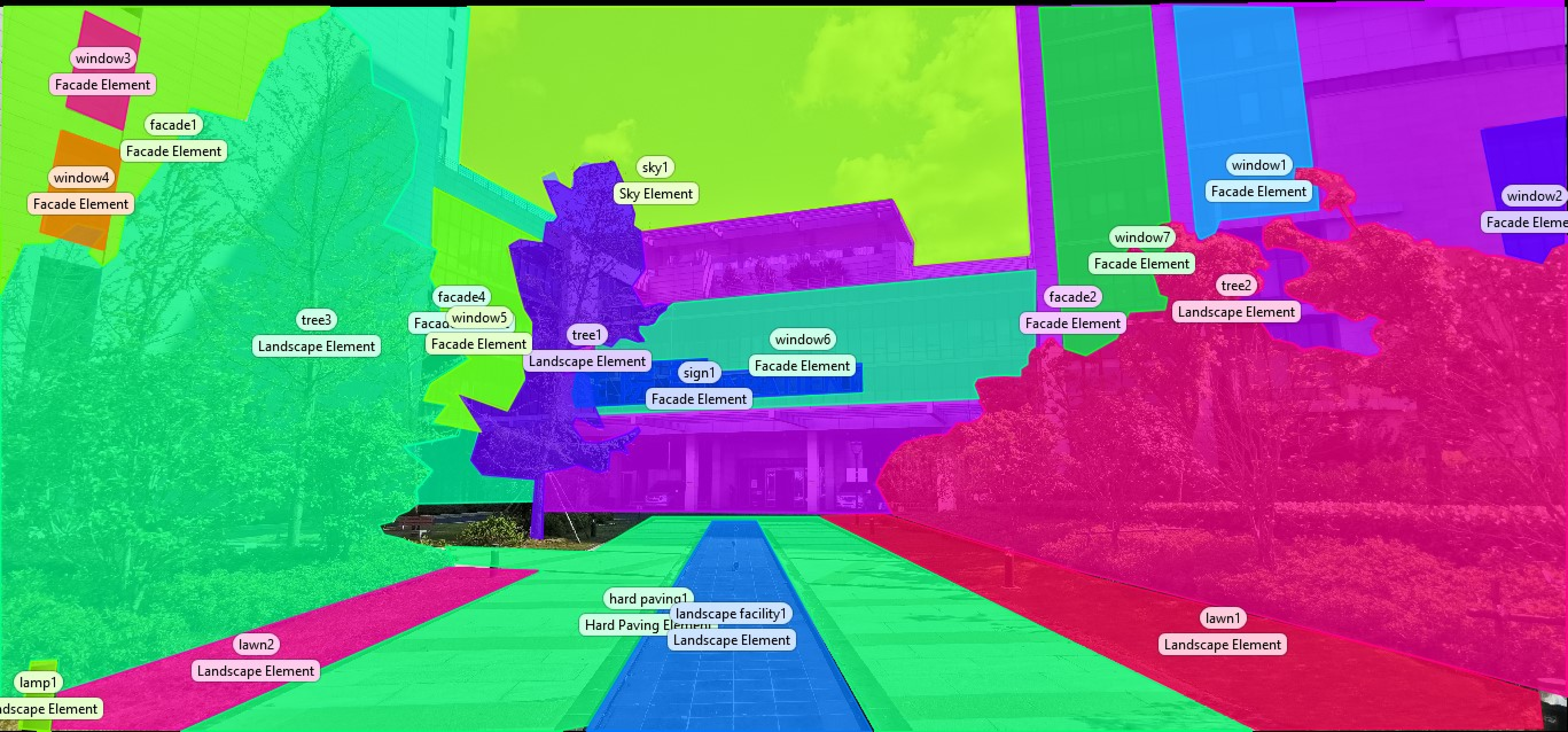
window3
Facade Element
facade1
Facade Element
window4
Facade Element
sky1
Sky Element
window1
Facade Element
window2
window7
Facade Element
tree2
Landscape Element
facade2
Facade Element
facade4
window5
Facade Element
tree3
Landscape Element
tree1
Landscape Element
sign1
Facade Element
window6
Facade Element
landscape facility1
Landscape Element
lawn1
Landscape Element
lawn2
Landscape Element
lamp1

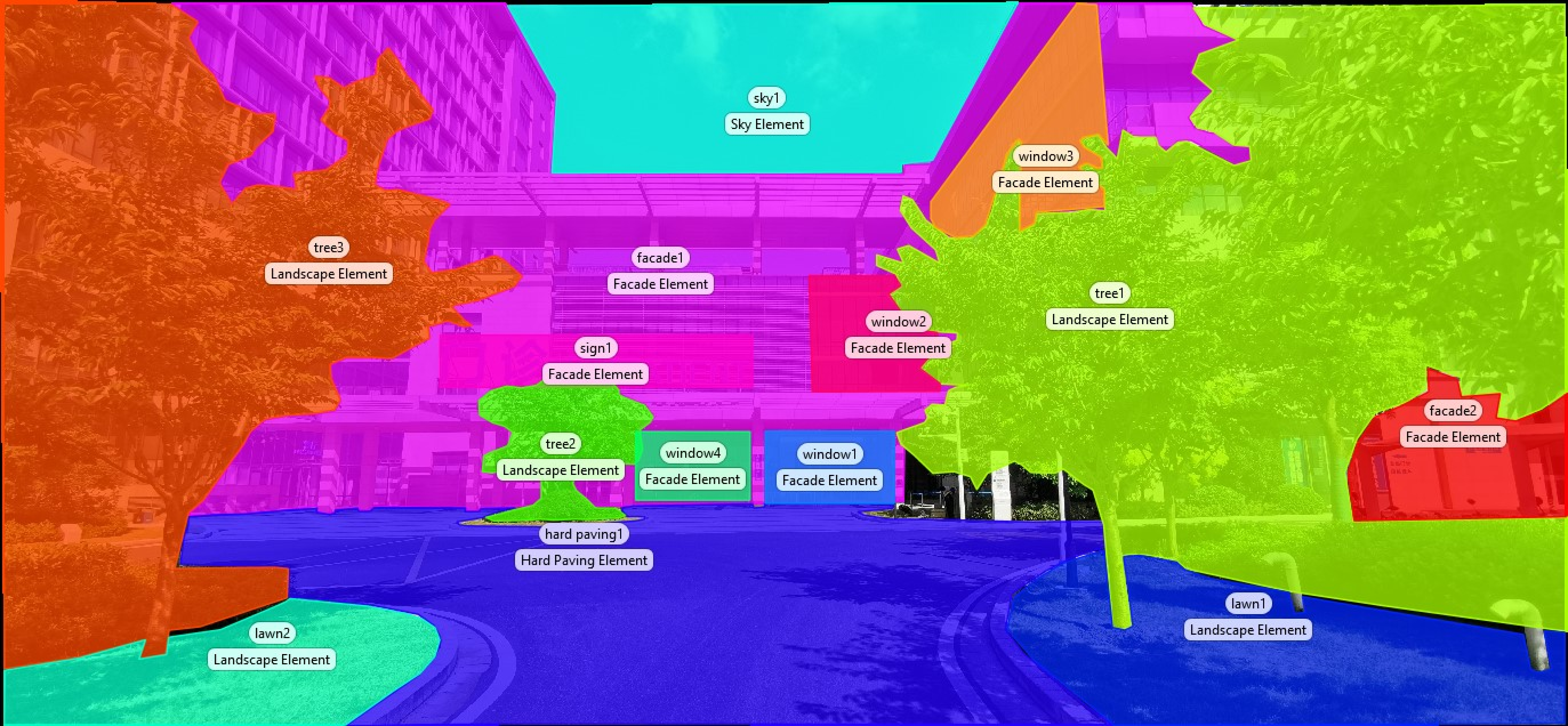
sky1
Sky Element
window3
Facade Element
tree3
Landscape Element
facade1
Facade Element
tree1
Landscape Element
window2
Facade Element
sign1
Facade Element
facade2
Facade Element
tree2
Landscape Element
window4
Facade Element
window1
Facade Element
hard paving1
Hard Paving Element
lawn1
Landscape Element
lawn2
Landscape Element

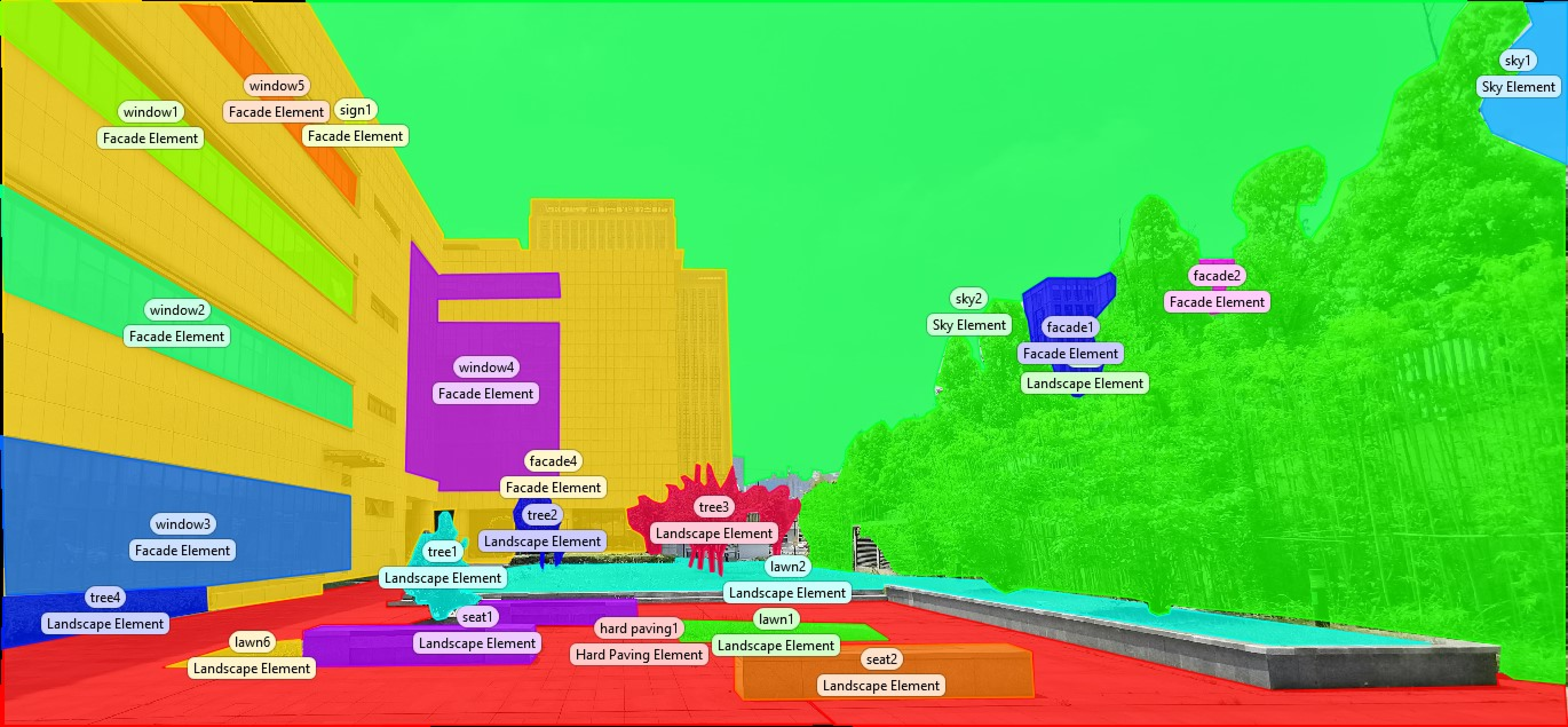
window5
Facade Element
window1
Facade Element
sign1
Facade Element
sky1
Sky Element
window2
Facade Element
sky2
Sky Element
facade2
Facade Element
facade1
Facade Element
Landscape Element
window4
Facade Element
facade4
Facade Element
tree3
Landscape Element
tree2
Landscape Element
window3
Facade Element
tree1
Landscape Element
lawn2
Landscape Element
tree4
Landscape Element
seat1
Landscape Element
hard paving1
Hard Paving Element
lawn1
Landscape Element
lawn6
Landscape Element
seat2
Landscape Element

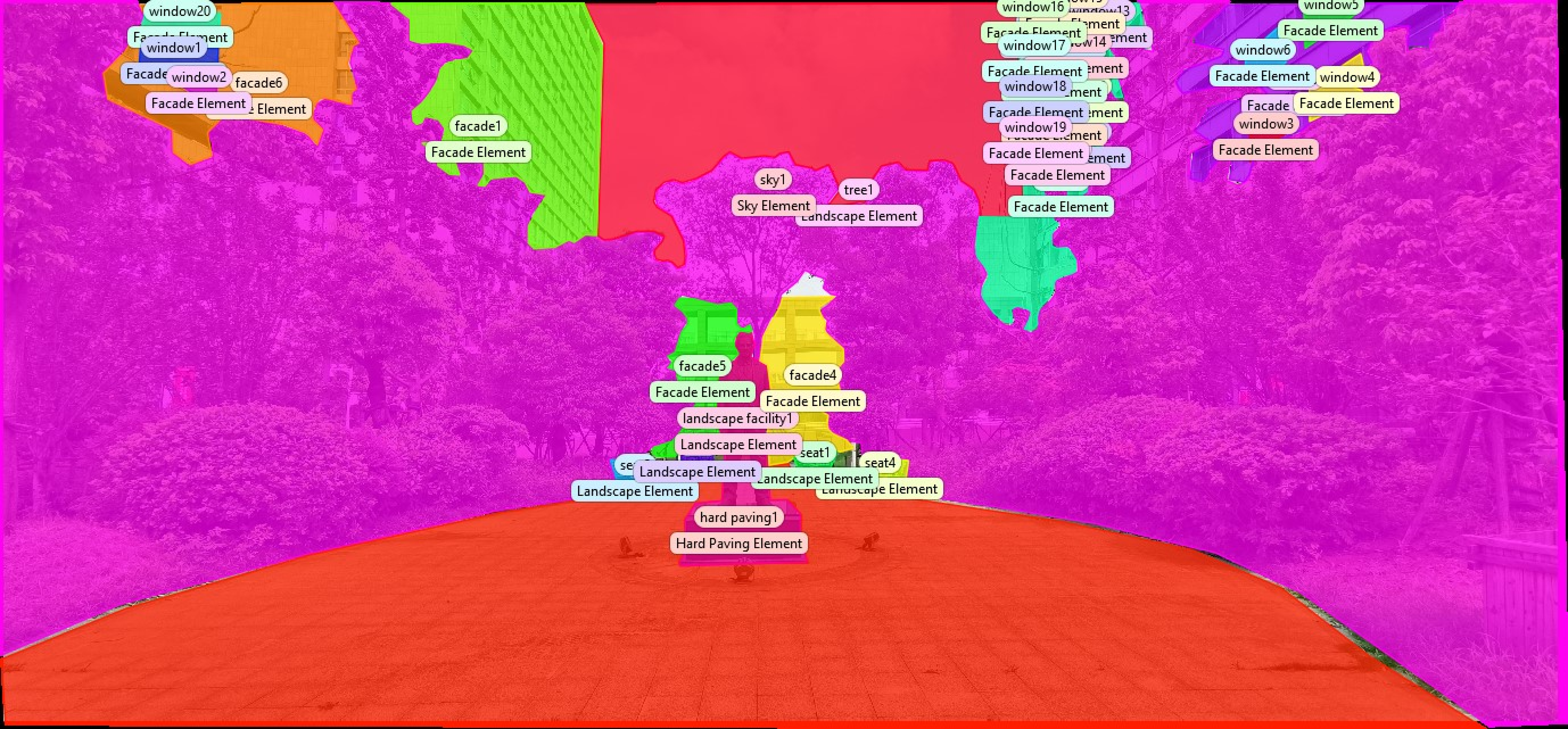
window20
Facade Element
window1
window2
facade6
Facade Element
facade1
Facade Element
sky1
Sky Element
tree1
window16
Facade Element
window17
Facade Element
window18
Facade Element
window19
Facade Element
Facade Element
Facade Element
window5
Facade Element
window6
Facade Element
window4
Facade Element
window3
Facade Element
facade5
Facade Element
facade4
Facade Element
landscape facility1
Landscape Element
seat1
seat4
Landscape Element
Landscape Element
hard paving1
Hard Paving Element

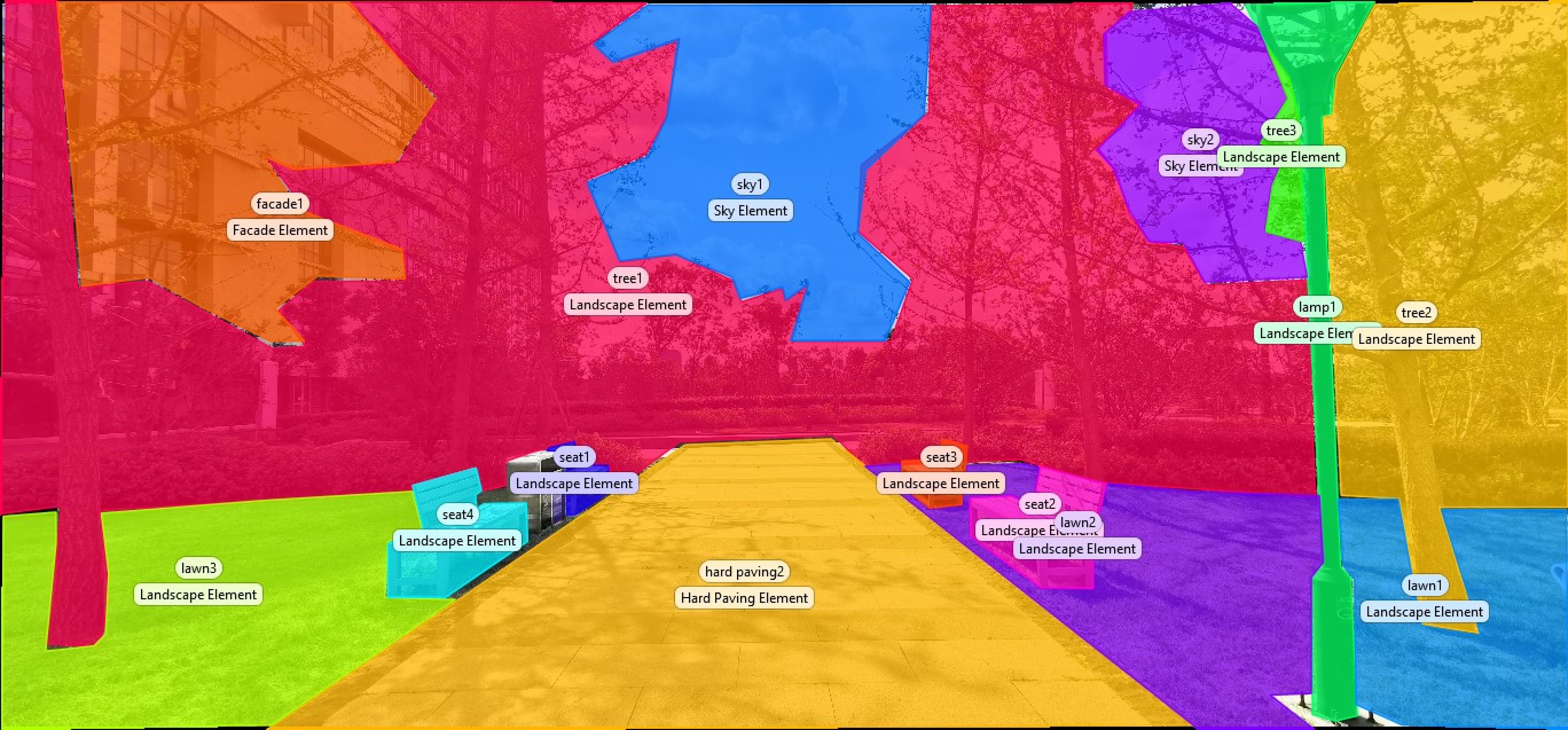
facade1
Facade Element
sky1
Sky Element
sky2
Sky Element
tree3
Landscape Element
tree1
Landscape Element
lamp1
Landscape Element
tree2
Landscape Element
seat1
Landscape Element
seat3
Landscape Element
seat2
Landscape Element
lawn2
Landscape Element
seat4
Landscape Element
lawn3
Landscape Element
hard paving2
Hard Paving Element
lawn1
Landscape Element

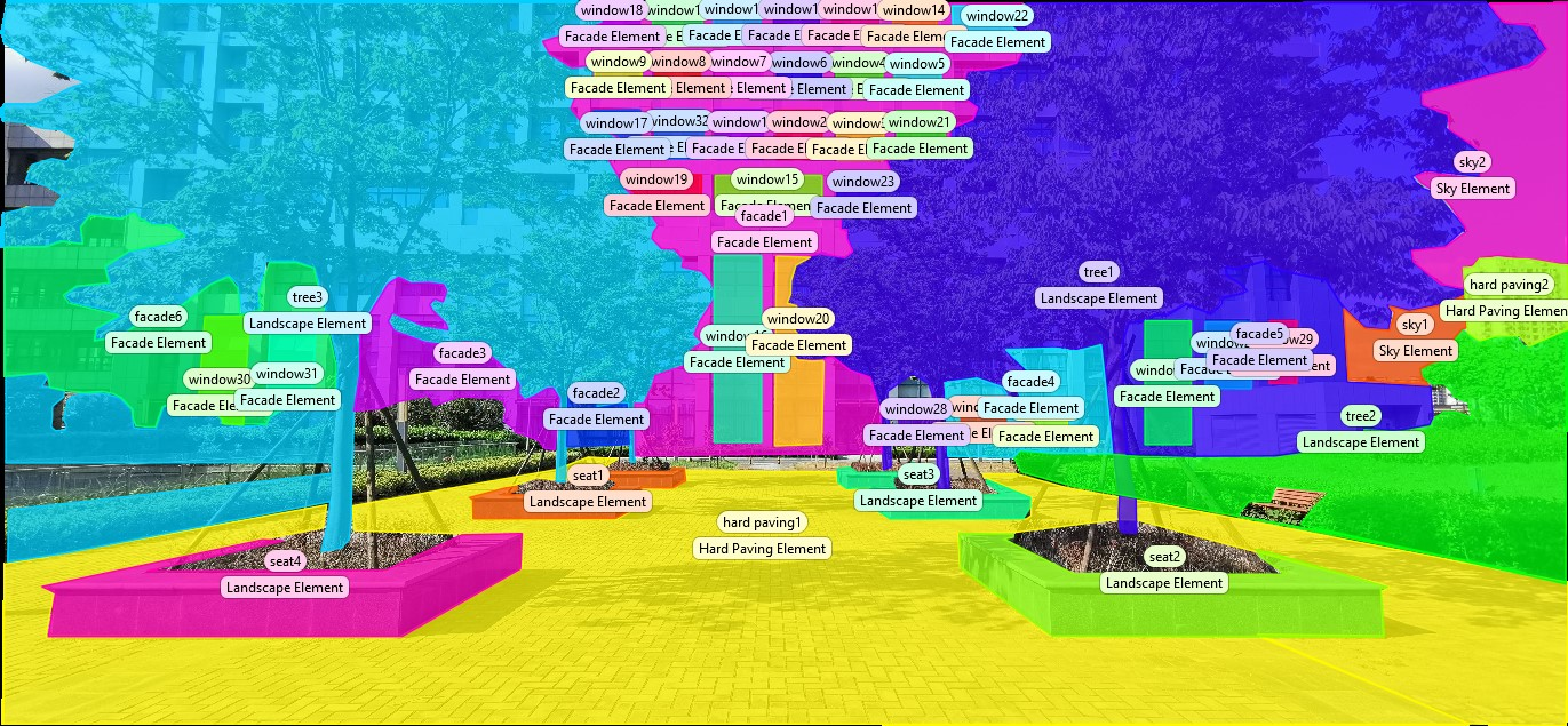

window18
window14
window22
Facade Element
window9
window8
window7
window6
window5
window17
window21
window19
window15
window23
facade1
tree1
Landscape Element
sky2
Sky Element
hard paving2
tree3
facade6
facade3
window20
facade5
sky1
window30
window31
facade2
facade4
window28
tree2
seat1
seat3
hard paving1
Hard Paving Element
seat4
seat2
